# Supplementary material for: Treatment options for Achilles tendinopathy: a scoping review of preclinical studies
Source: PeerJ. 2025 Jan 10;13:e18143. doi: 10.7717/peerj.18143 (PMC11727660; doi:10.7717/peerj.18143)
Supplement: Supplemental Information 1 [file peerj-13-18143-s001.docx]

**Treatment options for Achilles tendinopathy: A systematic review of preclinical studies**

**Appendix**

Authors with affiliations:

Drs. Nathanael Agyeman-Prempeh^1^

Dr. Huub Maas^2^

George L Burchell^3^

Prof. Neal L Millar^4^

Dr. Maarten Moen^5^

Prof. Dr. Ir. Theo Smit^6^

1. Amsterdam University Medical Centers, Dept of Orthopedic Surgery and Sports Medicine, Amsterdam Movement Sciences Research Institute **(n.agyeman-prempeh@amsterdamumc.nl)**

2. Department of Human Movement Sciences, Faculty of Behavioural and Movement Sciences, Movement Sciences, Vrije Universiteit Amsterdam, The Netherlands **(h.maas@vu.nl)**

3. Medical Library, Vrije Universiteit Amsterdam, Amsterdam, the Netherlands. (g.b.burchell@vu.nl)

4. Institute of Infection, Immunity and Inflammation, University of Glasgow, Glasgow, UK. (**Neal.Millar@glasgow.ac.uk)**

5. The Sport Physician Group, Department of Sports Medicine, OLVG, Amsterdam, the Netherlands; Department of Sports Medicine, Bergman Clinics, Naarden, the Netherlands) **(maarten.moen@nocnsf.nl)**

6. Amsterdam University Medical Centers, Dept of Orthopedic Surgery and Sports Medicine, Amsterdam Movement Sciences Research Institute **(**[**th.smit@amsterdamumc.nl**](mailto:th.smit@amsterdamumc.nl)**)**

Contents

[Appendix A – Components and cells of the Achilles tendon and their functions 3](#_Toc173160302)

[Appendix B - Search string 4](#_Toc173160303)

[Table 1: Search strategy in PubMed 4](#_Toc173160304)

[Table 2: Search strategy in Embase.com 7](#_Toc173160305)

[Table 3: Search strategy in Clarivate Analytics/Web of Science Core Collection 8](#_Toc173160306)

[Table 4: Search strategy in Cochrane 10](#_Toc173160307)

[Appendix C – SYRCLE Risk of bias assessment (Robvis tool) 12](#_Toc173160308)

[Appendix D – In vivo animal studies 15](#_Toc173160309)

[Appendix E – In vitro combined with in vivo animal studies 31](#_Toc173160310)

[Appendix F - In vitro studies 36](#_Toc173160311)

[References 37](#_Toc173160312)

**Abbreviations**

NR = Not reported

NA = Not applicable

ROB = Risk of bias

# Appendix A – Components and cells of the Achilles tendon and their functions

**non-collagenous ECM components**

| Proteoglycans |  |
| --- | --- |
| Decorin | Assists in the transfer of strain between discontinuous collagen fibrils and connect adjacent collagen fibrils |
| Aggrecan | Contributes to increase of tendon modulus and increases water volume in tendon for increased stiffness of the tendon |
| Versican | Links microfibrils to matrix components which contributes to structural properties |
| Collagen oligomeric matrix protein (COMP) | Influence of fiber sliding |
| Tenascin-C | Influences arrangement of fibers |
| Lubricin | Modulates tendon and fascicle sliding resistance |
| Biglycan | Bind to fibrillar collagen and may regulate fibrillogenesis |
| Fibromodulin and Lumican | Interact with fibrillar collagen and may inhibit fibril fusion |
| Elastin | Highly elastic protein which can extend more than 2 times its length and is located between the fascicles. Also, important for energy storage and preserving tendon structure after load. |
| Reference (5,6,7,9) |  |

**Collagenous ECM components**

| Collagenous ECM | Function and influence in tendon |
| --- | --- |
| Type I | Forms the greatest part of the tendon collagen and is parallel aligned. It has a stiff consistency with high mechanical strength |
| Type III | Plays role in the healing process and is more flexible than collagen type I. It produces smaller, less organized fibrils. |
| Type V | Infused in the collagen type I and plays role in fibrillogenesis and growth |
| Type XII | Ability to bind proteoglycans and type I fibrils and allow fibrils to slide when force is applied |
| Reference (5,6,7,9) |  |

**MMP and TIMP**

| MMP and TIMP cells | Function and influence in tendon |
| --- | --- |
| MMP-1 | Degrades collagen type I, II, III, VII, VIII, and X and is overexpressed in tendon injury. |
| MMP-2 | Degrades elastin, proteoglycans, and collagen type IV, V, VII, X, and XI. Is interactive with MMP 1 and overexpressed in tendon injury. |
| MMP-3 | Degrades proteoglycan, fibronectin, and collagen type II, IV, V, and IX and plays important role in the maintenance of tendon. Is downregulated in tendon injury. |
| MMP-8 | Degrades aggrecan and collagen type I, II and III |
| MMP-9 | Degrades collagen type IV, V, X, and XI and gelatin |
| MMP-12 | Degrades inter alia collagen type I and IV, elastin, aggrecan, and fibronectin |
| MMP-13 | Degrades collagen type I, II, and III and gelatin |
| TIMP-1 | Inhibits degeneration of extracellular matric |
| TIMP-2 | Downregulated in tendinopathy and tendon tears |
| Reference (5,6,7) |  |

**Inflammatory AT cells**

| Inflammatory cells | Function and influence in tendon |
| --- | --- |
| IL-1B | Reduced tendon strength and ECM degeneration, also ensure higher production of pro-inflammatory cells as a result of mechanical stretching. |
| IL-4 | Reduced tendon strength and boost acute inflammatory actions. |
| IL-6 | Increases the collagen synthesis and may deliver proinflammatory as anti-inflammatory actions |
| IL-10 | Anti-inflammatory properties and plays role in keeping normal tendon homeostasis. |
| IL-13 | Reduced tendon strength and boost acute inflammatory actions. And may play a role in ECM remodeling |
| COX-2 | Pro-inflammatory molecule |
| TNF-B | Reduced collagen type I expression and upregulates proinflammatory cytokines |
| PGE_2_ | Plays role in tenocyte proliferation and |
| Reference (9) |  |

# Appendix B - Search string

## Table 1: Search strategy in PubMed

| **Search** | **Query** | **Results** |
| --- | --- | --- |
| #5 | #4 NOT OLD PMIDs | 239 |
| #4 | Search: **#1 AND (#2 OR #3)** | 2,298 |
| #3 | Search: **"In Vitro Techniques"[Mesh] OR "Models, Biological"[Mesh] OR "Spheroids, Cellular"[Mesh] OR "Biological Assay"[Mesh] OR "Cell Culture Techniques"[Mesh] OR "ex vivo"[tiab] OR "model*"[tiab] OR "in vitro*"[tiab] OR "invitro*"[tiab] OR "assay*"[tiab] OR "tenoblast*"[tiab] OR "tenocyte*"[tiab]** | 6,448,224 |
| #2 | Search: **"animal experimentation"[MeSH Terms] OR "models, animal"[MeSH Terms] OR "invertebrates"[MeSH Terms] OR "Animals"[Mesh:noexp] OR "animal population groups"[MeSH Terms] OR "chordata"[MeSH Terms:noexp] OR "chordata, nonvertebrate"[MeSH Terms] OR "vertebrates"[MeSH Terms:noexp] OR "mammals"[MeSH Terms:noexp] OR "primates"[MeSH Terms:noexp] OR "artiodactyla"[MeSH Terms] OR "carnivora"[MeSH Terms] OR "cetacea"[MeSH Terms] OR "chiroptera"[MeSH Terms] OR "elephants"[MeSH Terms] OR "hyraxes"[MeSH Terms] OR "lagomorpha"[MeSH Terms] OR "marsupialia"[MeSH Terms] OR "monotremata"[MeSH Terms] OR "perissodactyla"[MeSH Terms] OR "rodentia"[MeSH Terms] OR "scandentia"[MeSH Terms] OR "sirenia"[MeSH Terms] OR "xenarthra"[MeSH Terms] OR "haplorhini"[MeSH Terms:noexp] OR "strepsirhini"[MeSH Terms] OR "platyrrhini"[MeSH Terms] OR "tarsii"[MeSH Terms] OR "catarrhini"[MeSH Terms:noexp] OR "cercopithecidae"[MeSH Terms] OR "hylobatidae"[MeSH Terms] OR "hominidae"[MeSH Terms:noexp] OR "gorilla gorilla"[MeSH Terms] OR "pan paniscus"[MeSH Terms] OR "pan troglodytes"[MeSH Terms] OR "pongo pygmaeus"[MeSH Terms] OR animals[tiab] OR animal[tiab] OR mice[tiab] OR mus[tiab] OR mouse[tiab] OR murine[tiab] OR woodmouse[tiab] OR rats[tiab] OR rat[tiab] OR murinae[tiab] OR muridae[tiab] OR cottonrat[tiab] OR cottonrats[tiab] OR hamster[tiab] OR hamsters[tiab] OR cricetinae[tiab] OR rodentia[tiab] OR rodent[tiab] OR rodents[tiab] OR "Rattus norvegicus"[tiab] OR pigs[tiab] OR pig[tiab] OR swine[tiab] OR swines[tiab] OR piglets[tiab] OR piglet[tiab] OR boar[tiab] OR boars[tiab] OR "sus scrofa"[tiab] OR ferrets[tiab] OR ferret[tiab] OR polecat[tiab] OR polecats[tiab] OR "mustela putorius"[tiab] OR "guinea pigs"[tiab] OR "guinea pig"[tiab] OR cavia[tiab] OR callithrix[tiab] OR marmoset[tiab] OR marmosets[tiab] OR cebuella[tiab] OR hapale[tiab] OR octodon[tiab] OR chinchilla[tiab] OR chinchillas[tiab] OR gerbillinae[tiab] OR gerbil[tiab] OR gerbils[tiab] OR jird[tiab] OR jirds[tiab] OR merione[tiab] OR meriones[tiab] OR rabbits[tiab] OR rabbit[tiab] OR hares[tiab] OR hare[tiab] OR diptera[tiab] OR flies[tiab] OR fly[tiab] OR dipteral[tiab] OR drosphila[tiab] OR drosophilidae[tiab] OR cats[tiab] OR cat[tiab] OR carus[tiab] OR felis[tiab] OR nematoda[tiab] OR nematode[tiab] OR nematoda[tiab] OR nematode[tiab] OR nematodes[tiab] OR sipunculida[tiab] OR dogs[tiab] OR dog[tiab] OR canine[tiab] OR canines[tiab] OR canis[tiab] OR sheep[tiab] OR sheeps[tiab] OR mouflon[tiab] OR mouflons[tiab] OR ovis[tiab] OR goats[tiab] OR goat[tiab] OR capra[tiab] OR capras[tiab] OR rupicapra[tiab] OR chamois[tiab] OR haplorhini[tiab] OR monkey[tiab] OR monkeys[tiab] OR anthropoidea[tiab] OR anthropoids[tiab] OR saguinus[tiab] OR tamarin[tiab] OR tamarins[tiab] OR leontopithecus[tiab] OR hominidae[tiab] OR ape[tiab] OR apes[tiab] OR pan[tiab] OR paniscus[tiab] OR "pan paniscus"[tiab] OR bonobo[tiab] OR bonobos[tiab] OR troglodytes[tiab] OR "pan troglodytes"[tiab] OR gibbon[tiab] OR gibbons[tiab] OR siamang[tiab] OR siamangs[tiab] OR nomascus[tiab] OR symphalangus[tiab] OR chimpanzee[tiab] OR chimpanzees[tiab] OR prosimians[tiab] OR "bush baby"[tiab] OR prosimian[tiab] OR bush babies[tiab] OR galagos[tiab] OR galago[tiab] OR pongidae[tiab] OR gorilla[tiab] OR gorillas[tiab] OR pongo[tiab] OR pygmaeus[tiab] OR "pongo pygmaeus"[tiab] OR orangutans[tiab] OR pygmaeus[tiab] OR lemur[tiab] OR lemurs[tiab] OR lemuridae[tiab] OR horse[tiab] OR horses[tiab] OR pongo[tiab] OR equus[tiab] OR cow[tiab] OR calf[tiab] OR bull[tiab] OR chicken[tiab] OR chickens[tiab] OR gallus[tiab] OR quail[tiab] OR bird[tiab] OR birds[tiab] OR quails[tiab] OR poultry[tiab] OR poultries[tiab] OR fowl[tiab] OR fowls[tiab] OR reptile[tiab] OR reptilia[tiab] OR reptiles[tiab] OR snakes[tiab] OR snake[tiab] OR lizard[tiab] OR lizards[tiab] OR alligator[tiab] OR alligators[tiab] OR crocodile[tiab] OR crocodiles[tiab] OR turtle[tiab] OR turtles[tiab] OR amphibian[tiab] OR amphibians[tiab] OR amphibia[tiab] OR frog[tiab] OR frogs[tiab] OR bombina[tiab] OR salientia[tiab] OR toad[tiab] OR toads[tiab] OR "epidalea calamita"[tiab] OR salamander[tiab] OR salamanders[tiab] OR eel[tiab] OR eels[tiab] OR fish[tiab] OR fishes[tiab] OR pisces[tiab] OR catfish[tiab] OR catfishes[tiab] OR siluriformes[tiab] OR arius[tiab] OR heteropneustes[tiab] OR sheatfish[tiab] OR perch[tiab] OR perches[tiab] OR percidae[tiab] OR perca[tiab] OR trout[tiab] OR trouts[tiab] OR char[tiab] OR chars[tiab] OR salvelinus[tiab] OR "fathead minnow"[tiab] OR minnow[tiab] OR cyprinidae[tiab] OR carps[tiab] OR carp[tiab] OR zebrafish[tiab] OR zebrafishes[tiab] OR goldfish[tiab] OR goldfishes[tiab] OR guppy[tiab] OR guppies[tiab] OR chub[tiab] OR chubs[tiab] OR tinca[tiab] OR barbels[tiab] OR barbus[tiab] OR pimephales[tiab] OR promelas[tiab] OR "poecilia reticulata"[tiab] OR mullet[tiab] OR mullets[tiab] OR seahorse[tiab] OR seahorses[tiab] OR mugil curema[tiab] OR atlantic cod[tiab] OR shark[tiab] OR sharks[tiab] OR catshark[tiab] OR anguilla[tiab] OR salmonid[tiab] OR salmonids[tiab] OR whitefish[tiab] OR whitefishes[tiab] OR salmon[tiab] OR salmons[tiab] OR sole[tiab] OR solea[tiab] OR "sea lamprey"[tiab] OR lamprey[tiab] OR lampreys[tiab] OR pumpkinseed[tiab] OR sunfish[tiab] OR sunfishes[tiab] OR tilapia[tiab] OR tilapias[tiab] OR turbot[tiab] OR turbots[tiab] OR flatfish[tiab] OR flatfishes[tiab] OR sciuridae[tiab] OR squirrel[tiab] OR squirrels[tiab] OR chipmunk[tiab] OR chipmunks[tiab] OR suslik[tiab] OR susliks[tiab] OR vole[tiab] OR voles[tiab] OR lemming[tiab] OR lemmings[tiab] OR muskrat[tiab] OR muskrats[tiab] OR lemmus[tiab] OR otter[tiab] OR otters[tiab] OR marten[tiab] OR martens[tiab] OR martes[tiab] OR weasel[tiab] OR badger[tiab] OR badgers[tiab] OR ermine[tiab] OR mink[tiab] OR minks[tiab] OR sable[tiab] OR sables[tiab] OR gulo[tiab] OR gulos[tiab] OR wolverine[tiab] OR wolverines[tiab] OR minks[tiab] OR mustela[tiab] OR llama[tiab] OR llamas[tiab] OR alpaca[tiab] OR alpacas[tiab] OR camelid[tiab] OR camelids[tiab] OR guanaco[tiab] OR guanacos[tiab] OR chiroptera[tiab] OR chiropteras[tiab] OR bat[tiab] OR bats[tiab] OR fox[tiab] OR foxes[tiab] OR iguana[tiab] OR iguanas[tiab] OR xenopus laevis[tiab] OR parakeet[tiab] OR parakeets[tiab] OR parrot[tiab] OR parrots[tiab] OR donkey[tiab] OR donkeys[tiab] OR mule[tiab] OR mules[tiab] OR zebra[tiab] OR zebras[tiab] OR shrew[tiab] OR shrews[tiab] OR bison[tiab] OR bisons[tiab] OR buffalo[tiab] OR buffaloes[tiab] OR deer[tiab] OR deers[tiab] OR bear[tiab] OR bears[tiab] OR panda[tiab] OR pandas[tiab] OR "wild hog"[tiab] OR "wild boar"[tiab] OR fitchew[tiab] OR fitch[tiab] OR beaver[tiab] OR beavers[tiab] OR jerboa[tiab] OR jerboas[tiab] OR capybara[tiab] OR capybaras[tiab] OR "in vivo*"[tiab] OR "invivo*"[tiab]** | 8,556,809 |
| #1 | Search: **"Achilles Tendon/injuries"[Mesh] OR ("Tendinopathy"[Mesh] AND "Achilles Tendon"[Mesh]) OR "achilles tendinopath*"[tiab] OR "achillodyn*"[tiab] OR ( ("achilles"[tiab] OR "calcaneal"[tiab]) AND ("tendinitis*"[tiab] OR "tendinopath*"[tiab] OR "tendinosis*"[tiab] OR "tendonitis*"[tiab] OR "tendon-patholog*"[tiab] OR "pain*"[tiab] OR "injur*"[tiab] OR "paratendonitis"[tiab] OR "tenosynovitis"[tiab]) )** | 9,585 |

## Table 2: Search strategy in Embase.com

| **Search** | **Query** | **Results** |
| --- | --- | --- |
| #6 | #5 NOT PUIs | 238 |
| #5 | #4 NOT ('chapter'/it OR 'conference abstract'/it OR 'conference paper'/it OR 'conference review'/it OR 'editorial'/it OR 'erratum'/it OR 'letter'/it OR 'note'/it OR 'short survey'/it OR 'tombstone'/it) | 1,744 |
| #4 | #1 AND (#2 OR #3) | 2,268 |
| #3 | 'in vitro study'/exp OR 'multicellular spheroid'/exp OR 'cell culture technique'/exp OR (‘in vitro’ OR ‘ex vivo’ OR ‘model*’ OR ‘in vitro*’ OR ‘invitro*’ OR ‘assay*’ OR ‘tenoblast*’ OR ‘tenocyte*’):ti,ab,kw | 11,642,891 |
| #2 | 'animal experiment'/exp OR 'animal model'/exp OR (animals OR animal OR mice OR mus OR mouse OR murine OR woodmouse OR rats OR rat OR murinae OR muridae OR cottonrat OR cottonrats OR hamster OR hamsters OR cricetinae OR rodentia OR rodent OR rodents OR ‘Rattus norvegicus’ OR pigs OR pig OR swine OR swines OR piglets OR piglet OR boar OR boars OR ‘sus scrofa’ OR ferrets OR ferret OR polecat OR polecats OR ‘mustela putorius’ OR ‘guinea pigs’ OR ‘guinea pig’ OR cavia OR callithrix OR marmoset OR marmosets OR cebuella OR hapale OR octodon OR chinchilla OR chinchillas OR gerbillinae OR gerbil OR gerbils OR jird OR jirds OR merione OR meriones OR rabbits OR rabbit OR hares OR hare OR diptera OR flies OR fly OR dipteral OR drosphila OR drosophilidae OR cats OR cat OR carus OR felis OR nematoda OR nematode OR nematoda OR nematode OR nematodes OR sipunculida OR dogs OR dog OR canine OR canines OR canis OR sheep OR sheeps OR mouflon OR mouflons OR ovis OR goats OR goat OR capra OR capras OR rupicapra OR chamois OR haplorhini OR monkey OR monkeys OR anthropoidea OR anthropoids OR saguinus OR tamarin OR tamarins OR leontopithecus OR hominidae OR ape OR apes OR pan OR paniscus OR ‘pan paniscus’ OR bonobo OR bonobos OR troglodytes OR ‘pan troglodytes’ OR gibbon OR gibbons OR siamang OR siamangs OR nomascus OR symphalangus OR chimpanzee OR chimpanzees OR prosimians OR ‘bush baby’ OR prosimian OR bush babies OR galagos OR galago OR pongidae OR gorilla OR gorillas OR pongo OR pygmaeus OR ‘pongo pygmaeus’ OR orangutans OR pygmaeus OR lemur OR lemurs OR lemuridae OR horse OR horses OR pongo OR equus OR cow OR calf OR bull OR chicken OR chickens OR gallus OR quail OR bird OR birds OR quails OR poultry OR poultries OR fowl OR fowls OR reptile OR reptilia OR reptiles OR snakes OR snake OR lizard OR lizards OR alligator OR alligators OR crocodile OR crocodiles OR turtle OR turtles OR amphibian OR amphibians OR amphibia OR frog OR frogs OR bombina OR salientia OR toad OR toads OR ‘epidalea calamita’ OR salamander OR salamanders OR eel OR eels OR fish OR fishes OR pisces OR catfish OR catfishes OR siluriformes OR arius OR heteropneustes OR sheatfish OR perch OR perches OR percidae OR perca OR trout OR trouts OR char OR chars OR salvelinus OR ‘fathead minnow’ OR minnow OR cyprinidae OR carps OR carp OR zebrafish OR zebrafishes OR goldfish OR goldfishes OR guppy OR guppies OR chub OR chubs OR tinca OR barbels OR barbus OR pimephales OR promelas OR ‘poecilia reticulata’ OR mullet OR mullets OR seahorse OR seahorses OR mugil curema OR atlantic cod OR shark OR sharks OR catshark OR anguilla OR salmonid OR salmonids OR whitefish OR whitefishes OR salmon OR salmons OR sole OR solea OR ‘sea lamprey’ OR lamprey OR lampreys OR pumpkinseed OR sunfish OR sunfishes OR tilapia OR tilapias OR turbot OR turbots OR flatfish OR flatfishes OR sciuridae OR squirrel OR squirrels OR chipmunk OR chipmunks OR suslik OR susliks OR vole OR voles OR lemming OR lemmings OR muskrat OR muskrats OR lemmus OR otter OR otters OR marten OR martens OR martes OR weasel OR badger OR badgers OR ermine OR mink OR minks OR sable OR sables OR gulo OR gulos OR wolverine OR wolverines OR minks OR mustela OR llama OR llamas OR alpaca OR alpacas OR camelid OR camelids OR guanaco OR guanacos OR chiroptera OR chiropteras OR bat OR bats OR fox OR foxes OR iguana OR iguanas OR xenopus laevis OR parakeet OR parakeets OR parrot OR parrots OR donkey OR donkeys OR mule OR mules OR zebra OR zebras OR shrew OR shrews OR bison OR bisons OR buffalo OR buffaloes OR deer OR deers OR bear OR bears OR panda OR pandas OR ‘wild hog’ OR ‘wild boar’ OR fitchew OR fitch OR beaver OR beavers OR jerboa OR jerboas OR capybara OR capybaras OR ‘in vivo*’ OR ‘invivo*’):ti,ab,kw | 4,107,993 |
| #1 | 'achilles tendinitis'/exp OR (‘achilles tendinopath*’ OR ‘achillodyn*’ OR ((‘achilles’ OR ‘calcaneal’)  AND (‘tendinitis*’ OR ‘tendinopath*’ OR ‘tendinosis*’ OR ‘tendonitis*’ OR ‘tendon-patholog*’ OR ‘pain*’ OR ‘injur*’ OR ‘paratendonitis’ OR ‘tenosynovitis’))):ti,ab,kw | 10,245 |

## Table 3: Search strategy in Clarivate Analytics/Web of Science Core Collection

| **Search** | **Query** | **Results** |
| --- | --- | --- |
| #5 | #4 NOT OLD WOFS | 51 |
| #4 | #1 AND (#2 OR #3) | 3,337 |
| #3 | TS=(“ex vivo” OR "model*" OR "in vitro*" OR "invitro*" OR "assay*" OR “tenoblast*” OR “tenocyte*”) | 12,222,336 |
| #2 | TS=(animals OR animal OR mice OR mus OR mouse OR murine OR woodmouse OR rats OR rat OR murinae OR muridae OR cottonrat OR cottonrats OR hamster OR hamsters OR cricetinae OR rodentia OR rodent OR rodents OR “Rattus norvegicus” OR pigs OR pig OR swine OR swines OR piglets OR piglet OR boar OR boars OR "sus scrofa" OR ferrets OR ferret OR polecat OR polecats OR "mustela putorius" OR "guinea pigs" OR "guinea pig" OR cavia OR callithrix OR marmoset OR marmosets OR cebuella OR hapale OR octodon OR chinchilla OR chinchillas OR gerbillinae OR gerbil OR gerbils OR jird OR jirds OR merione OR meriones OR rabbits OR rabbit OR hares OR hare OR diptera OR flies OR fly OR dipteral OR drosphila OR drosophilidae OR cats OR cat OR carus OR felis OR nematoda OR nematode OR nematoda OR nematode OR nematodes OR sipunculida OR dogs OR dog OR canine OR canines OR canis OR sheep OR sheeps OR mouflon OR mouflons OR ovis OR goats OR goat OR capra OR capras OR rupicapra OR chamois OR haplorhini OR monkey OR monkeys OR anthropoidea OR anthropoids OR saguinus OR tamarin OR tamarins OR leontopithecus OR hominidae OR ape OR apes OR pan OR paniscus OR "pan paniscus" OR bonobo OR bonobos OR troglodytes OR "pan troglodytes" OR gibbon OR gibbons OR siamang OR siamangs OR nomascus OR symphalangus OR chimpanzee OR chimpanzees OR prosimians OR "bush baby" OR prosimian OR bush babies OR galagos OR galago OR pongidae OR gorilla OR gorillas OR pongo OR pygmaeus OR "pongo pygmaeus" OR orangutans OR pygmaeus OR lemur OR lemurs OR lemuridae OR horse OR horses OR pongo OR equus OR cow OR calf OR bull OR chicken OR chickens OR gallus OR quail OR bird OR birds OR quails OR poultry OR poultries OR fowl OR fowls OR reptile OR reptilia OR reptiles OR snakes OR snake OR lizard OR lizards OR alligator OR alligators OR crocodile OR crocodiles OR turtle OR turtles OR amphibian OR amphibians OR amphibia OR frog OR frogs OR bombina OR salientia OR toad OR toads OR "epidalea calamita" OR salamander OR salamanders OR eel OR eels OR fish OR fishes OR pisces OR catfish OR catfishes OR siluriformes OR arius OR heteropneustes OR sheatfish OR perch OR perches OR percidae OR perca OR trout OR trouts OR char OR chars OR salvelinus OR "fathead minnow" OR minnow OR cyprinidae OR carps OR carp OR zebrafish OR zebrafishes OR goldfish OR goldfishes OR guppy OR guppies OR chub OR chubs OR tinca OR barbels OR barbus OR pimephales OR promelas OR "poecilia reticulata" OR mullet OR mullets OR seahorse OR seahorses OR mugil curema OR atlantic cod OR shark OR sharks OR catshark OR anguilla OR salmonid OR salmonids OR whitefish OR whitefishes OR salmon OR salmons OR sole OR solea OR "sea lamprey" OR lamprey OR lampreys OR pumpkinseed OR sunfish OR sunfishes OR tilapia OR tilapias OR turbot OR turbots OR flatfish OR flatfishes OR sciuridae OR squirrel OR squirrels OR chipmunk OR chipmunks OR suslik OR susliks OR vole OR voles OR lemming OR lemmings OR muskrat OR muskrats OR lemmus OR otter OR otters OR marten OR martens OR martes OR weasel OR badger OR badgers OR ermine OR mink OR minks OR sable OR sables OR gulo OR gulos OR wolverine OR wolverines OR minks OR mustela OR llama OR llamas OR alpaca OR alpacas OR camelid OR camelids OR guanaco OR guanacos OR chiroptera OR chiropteras OR bat OR bats OR fox OR foxes OR iguana OR iguanas OR xenopus laevis OR parakeet OR parakeets OR parrot OR parrots OR donkey OR donkeys OR mule OR mules OR zebra OR zebras OR shrew OR shrews OR bison OR bisons OR buffalo OR buffaloes OR deer OR deers OR bear OR bears OR panda OR pandas OR "wild hog" OR "wild boar" OR fitchew OR fitch OR beaver OR beavers OR jerboa OR jerboas OR capybara OR capybaras OR "in vivo*" OR "invivo*") | 8,853,535 |
| #1 | TS=(“achilles tendinopath*” OR “achillodyn*” OR ((“achilles” OR “calcaneal”) AND (“tendinitis*” OR “tendinopath*” OR “tendinosis*” OR “tendonitis*” OR “tendon-patholog*” OR “pain*” OR “injur*” OR “paratendonitis” OR “tenosynovitis”))) | 9,512 |

## Table 4: Search strategy in Cochrane

| **Search** | **Query** | **Results** |
| --- | --- | --- |
| #5 | #4 NOT OLD COCHRANE | 36 |
| #4 | #1 AND (#2 OR #3) | 192 |
| #3 | (“ex vivo” OR  "model*" OR  "in vitro*" OR  "invitro*" OR  "assay*" OR  “tenoblast*” OR  “tenocyte*”):ti,ab,kw | 157591 |
| #2 | (animals OR animal OR mice OR mus OR mouse OR murine OR woodmouse OR rats OR rat OR murinae OR muridae OR cottonrat OR cottonrats OR hamster OR hamsters OR cricetinae OR rodentia OR rodent OR rodents OR “Rattus norvegicus” OR pigs OR pig OR swine OR swines OR piglets OR piglet OR boar OR boars OR "sus scrofa" OR ferrets OR ferret OR polecat OR polecats OR "mustela putorius" OR "guinea pigs" OR "guinea pig" OR cavia OR callithrix OR marmoset OR marmosets OR cebuella OR hapale OR octodon OR chinchilla OR chinchillas OR gerbillinae OR gerbil OR gerbils OR jird OR jirds OR merione OR meriones OR rabbits OR rabbit OR hares OR hare OR diptera OR flies OR fly OR dipteral OR drosphila OR drosophilidae OR cats OR cat OR carus OR felis OR nematoda OR nematode OR nematoda OR nematode OR nematodes OR sipunculida OR dogs OR dog OR canine OR canines OR canis OR sheep OR sheeps OR mouflon OR mouflons OR ovis OR goats OR goat OR capra OR capras OR rupicapra OR chamois OR haplorhini OR monkey OR monkeys OR anthropoidea OR anthropoids OR saguinus OR tamarin OR tamarins OR leontopithecus OR hominidae OR ape OR apes OR pan OR paniscus OR "pan paniscus" OR bonobo OR bonobos OR troglodytes OR "pan troglodytes" OR gibbon OR gibbons OR siamang OR siamangs OR nomascus OR symphalangus OR chimpanzee OR chimpanzees OR prosimians OR "bush baby" OR prosimian OR bush babies OR galagos OR galago OR pongidae OR gorilla OR gorillas OR pongo OR pygmaeus OR "pongo pygmaeus" OR orangutans OR pygmaeus OR lemur OR lemurs OR lemuridae OR horse OR horses OR pongo OR equus OR cow OR calf OR bull OR chicken OR chickens OR gallus OR quail OR bird OR birds OR quails OR poultry OR poultries OR fowl OR fowls OR reptile OR reptilia OR reptiles OR snakes OR snake OR lizard OR lizards OR alligator OR alligators OR crocodile OR crocodiles OR turtle OR turtles OR amphibian OR amphibians OR amphibia OR frog OR frogs OR bombina OR salientia OR toad OR toads OR "epidalea calamita" OR salamander OR salamanders OR eel OR eels OR fish OR fishes OR pisces OR catfish OR catfishes OR siluriformes OR arius OR heteropneustes OR sheatfish OR perch OR perches OR percidae OR perca OR trout OR trouts OR char OR chars OR salvelinus OR "fathead minnow" OR minnow OR cyprinidae OR carps OR carp OR zebrafish OR zebrafishes OR goldfish OR goldfishes OR guppy OR guppies OR chub OR chubs OR tinca OR barbels OR barbus OR pimephales OR promelas OR "poecilia reticulata" OR mullet OR mullets OR seahorse OR seahorses OR mugil curema OR atlantic cod OR shark OR sharks OR catshark OR anguilla OR salmonid OR salmonids OR whitefish OR whitefishes OR salmon OR salmons OR sole OR solea OR "sea lamprey" OR lamprey OR lampreys OR pumpkinseed OR sunfish OR sunfishes OR tilapia OR tilapias OR turbot OR turbots OR flatfish OR flatfishes OR sciuridae OR squirrel OR squirrels OR chipmunk OR chipmunks OR suslik OR susliks OR vole OR voles OR lemming OR lemmings OR muskrat OR muskrats OR lemmus OR otter OR otters OR marten OR martens OR martes OR weasel OR badger OR badgers OR ermine OR mink OR minks OR sable OR sables OR gulo OR gulos OR wolverine OR wolverines OR minks OR mustela OR llama OR llamas OR alpaca OR alpacas OR camelid OR camelids OR guanaco OR guanacos OR chiroptera OR chiropteras OR bat OR bats OR fox OR foxes OR iguana OR iguanas OR xenopus laevis OR parakeet OR parakeets OR parrot OR parrots OR donkey OR donkeys OR mule OR mules OR zebra OR zebras OR shrew OR shrews OR bison OR bisons OR buffalo OR buffaloes OR deer OR deers OR bear OR bears OR panda OR pandas OR "wild hog" OR "wild boar" OR fitchew OR fitch OR beaver OR beavers OR jerboa OR jerboas OR capybara OR capybaras OR "in vivo*" OR "invivo*"):ti,ab,kw | 86,947 |

Appendix C – SYRCLE Risk of bias assessment (Robvis tool)


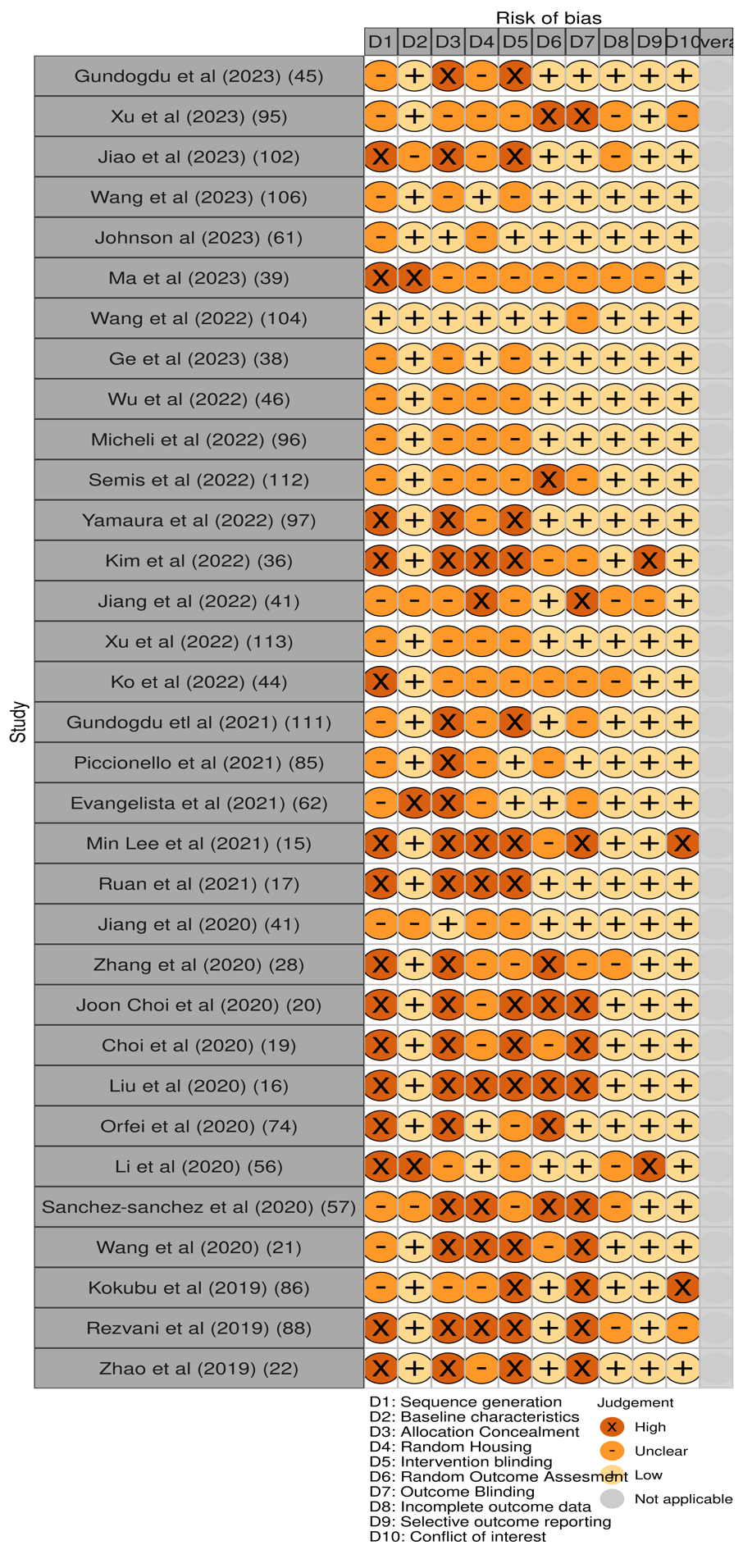


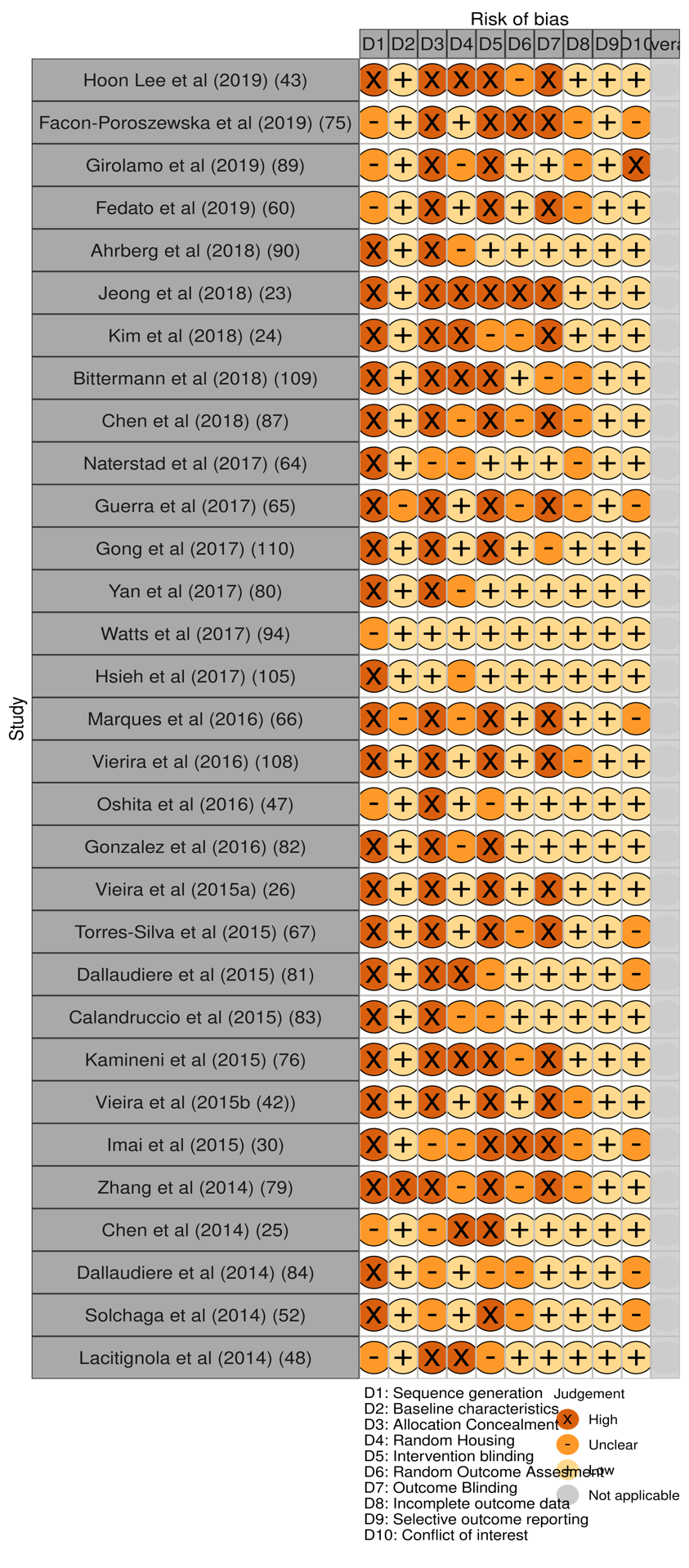


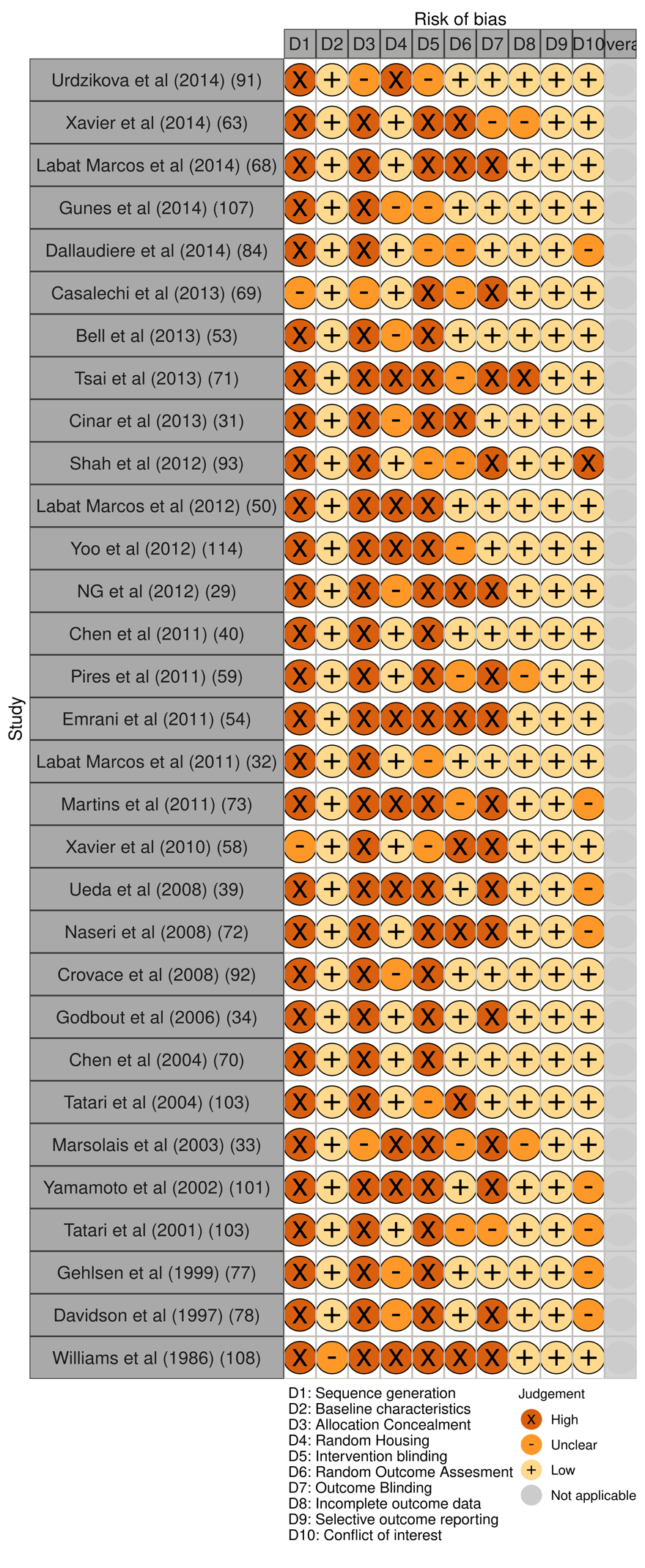


# Appendix D – In vivo animal studies

| Author & Year  Study aim  Reference | No. of Included animals  Animal breed  Assigned groups | Time of analysis | Method of inducting AT | Treatment evaluated | Histological outcome | Biochemical outcome | Biomechanical outcome |
| --- | --- | --- | --- | --- | --- | --- | --- |
| Gundogdu et al. (2023) (45)  Analyzing the effect of combining treatments | 50 12-16 weeks old male Wistar albino rats  Groups (n=10 per group):  - Control (C)  - AT group (injury model)  - AT+ Exercise  -AT+Omega 3  -AT+Exercise+Omega3 | After 9 weeks | Injection with collagenase type I 30uL | The exercise protocol was carried out on a rodent treadmill (Commat, Turkey). Performed 5 days a week, 20 m/min speed, 0-degree incline and 30 min/day for 8 weeks.  Omega-3 (150 mg/kg DHA + 250 mg/kg EPA) was given by oral gavage for 9 weeks (5 days/week), one week for adaptation and the other 8 weeks for exercise protocol | AT+Exercise+Omega3 group compared to other groups  -More new capillary vessel structures and fibrocytes and fibroblasts  - Less degeneration and inflammation | AT+Exercise+Omega3 group compered to AT group  -Statistically significant lower expressions of -Decreased expression of TNF-B1, MMP-13 and IL-1B  - Lower oxidative stress index | AT+Exercise+Omega3 group compered to AT group  -Higher ultimate tensile force, yield force and stiffness |
| Johnson et al.  (2023) (61)  Comparing the efficacy of treatment | 35 male mice (C57BL/6J), 8 weeks of age.  Groups (n=5 per group):  -Rest (R)  -Slow-flat (20cm/s, 0-degree incline) (SF)  -Fast-flat (32cm/s, 0-degree incline) (FF)  -Slow-incline (20cm/s, 17-degree incline) (SI)  -Fast-incline (32cm/s, 17-degree incline) (FI) | After 2 weeks | Injection with 6 uL of 100 ng active human recombinant TGF-β1 in right Achilles tendon | Exercise protocols consisted of 25 min/day starting on day 3 of the experimental protocol for 5 days per week with 2 days of rest each week on treadmill for 2 weeks  -Slow-flat (20cm/s, 0-degree incline) (SF)  -Fast-flat (32cm/s, 0-degree incline) (FF)  -Slow-incline (20cm/s, 17-degree incline) (SI)  -Fast-incline (32cm/s, 17-degree incline) (FI) | The results were compared with the contralateral uninjured limb  Fast-flat  -Increased tendon mid substance chondroid metaplasia  -Minor peritenon hyperplasia  -Bursal synovial hyperplasia and increased perivascular mononuclear cells | The results were compared with the contralateral uninjured limb  Slow-flat  -Lowest CXM expression  Fast and slow incline  -Highest CXM serum expression  CXM= Collagenase 10 breakdown product | NR |
| Ma et al.  (2023) (39)  Comparing and analyzing the efficacy of treatment | New Zealand rabbits (No amount reported)  Groups:  -Control  -Tendinopathy  -hHF-MSCs group  -Umbilical cord MSC group (UCMSC) | After 48 days | Injection with collagenase type I 2400 U/2 mL | Four injections with Human hair follicle-derived mesenchymal stem cells (hHF-MSCs).  First 3 injections at 72-hours intervals and last injection 10 days before sacrifice | hHF-MSCs group compared to AT group  -Better ordered collagen fibers  -Less inflammatory cells  -Normal amount of prokaryotic cells  hHF-MSCs group compared to UCMSC group  -No obvious fiber necrosis  -No inflammatory cells around blood vessels | hHF-MSCs group compared to AT group  -Higher expression of collagen I and III  -Higher expression of TNC mRNA and protein levels  -Lower expression of MMP-9 mRNA and protein levels | hHF-MSCs group and UCMSC group compared to AT group  -Upregulated maximum load |
| Wang et al.  (2023) (106)  Analyzing the efficacy of treatment | 113 Male Sprague Dawley rats (8 weeks old, 200-250g)  Groups:  -Control (n=27)  -Sham (n=43)  -Irreversible Electroporation (IRE) (n+43) | After 77 days | Injection with collagenase type I 12mg/mL 25uL | Irreversible Electroporation.  The suspended cells were exposed to a voltage protocol (100-ms monopolar pulses, interpulse interval of 100 ms) of different electric field intensities (625, 750, 875, 1000, 1250, and 1500 V/cm) and pulse numbers (60, 90, and 180 pulses), which were controlled by an ECM830 square-wave electro- poration system (BTX).  Treatment done on 21, 1, 7, 21, 35, 49, and 77 days after induction of AT | IRE compared to sham group  -Only some pyknotic abnormal nuclei left  -Higher number of fibroblasts and microvascular lumens  -Higher cell proliferation  -More spindle shaped tenocytes parallel to the collagen fibers  -Less disorganized collagen fibers | IRE compared to sham group  -Higher expression of caspase-3 and PGE_2_  - Higher expression of TNMD-positive cells  - Higher proliferative activity  -Lower CD31 expression  -Lower CGRP expression | IRE compared to sham group  -Higher maximum load  -Higher recovery of maximum load  -Lower stiffness  -Higher tensile load  IRE compared to control group  -Comparable recovery of maximum load  -Comparable stiffness |
| Ge et al.  (2023) (38)  Comparing the efficacy of treatments | 110 Male 8-weeks old Sprague-Dawley rats  groups (n=22 per group):  -Control health group (no AT)  -PBS(Saline) group+AT  -DNA Gel group+AT  -TSPCs group+AT  -TSPCs/gel group+AT | After 4 and 8 weeks | Injection with collagenase type I 25uL | Injection with Tendon stem progenitor cells (TSCPS) incapsulated in DNA hydrogel (TSPCs-Gel). Injection with only DNA-gel or TSCPS cells. | The alignment of collagen in the TSPCs-Gel group was improved, and the number of round-shaped nuclei had dropped to the normal level of the healthy tendon after 8 weeks of treatment  TSPCs/gel group compared to TSPCs and Saline group  Higher modified Stoll scores | TSPCs/gel group compared to TSPCs and Saline group  -Significantly higher expression of collagen type I and tenomodulin  -Decreased expression of collagen type III | TSPCs/gel group compared to TSPCs and Saline group  -Higher load to failure, elastic modulus and stiffness.  -Lower cross-sectional area |
| Wang et al.  (2022) (104)  Analyzing the efficacy of treatment | 48 adult male New Zealand white rabbits  Groups:  -Prostaglandin E2(AT)(n=32)  -Saline (n=8)  -Normal(n=8)  Prostaglandin group subdivided (n=8/group);  - Asperosaponin VI 0 mg/kg (AT)  - Asperosaponin VI 10 mg/kg  - Asperosaponin VI 20 mg/kg  - Asperosaponin VI 40 mg/kg | After 4 weeks | Injection with 300 ng of prostaglandin E | Intraperitoneal injections of 10, 20 or  40 mg/kg Asperosaponin VI dissolved in saline or saline only once a day for 4 weeks. | 40 mg/KG group compared to AT group  -Tendon fibers arranged in continuous and more arranged manner  -Normal cell densities  The tendon damage scores in the 10, 20 and 40 mg/kg Asperosaponin VI groups decreased successively and were significantly  lower than those in the model group | 20 and 40 mg/KG group compared to AT group  -Downregulated MMP1  10, 20 and 40 mg/KG group compared to AT group  -Upregulated TGFB1, Collagen 1 and TNMD  10 and 20 mg/KG group compared to AT group  -Downregulated collagen 3 expression | NR |
| Semis et al.  (2022) (112)  Comparing the efficacy of treatment | 35 10 to 12 weeks old Sprague-Dawley rats  Groups (n=7/group):  -Control (C)  -Tendinopathy (AT)  -Diclofenac (D)  -Quercetin 25 mg/kg (Q25)  -Quercetin 50 mg/kg (Q50) | After 7 days | Injection with collagenase type I 10mg/mL 10uL | Oral diclofenac administration 75mg/3ML  Quercetin was administered orally at doses of 25 or 50 mg/kg twice a day for 7 days | NR | In all test higher dose of Quercetin (Q50) was more effective than (Q25) unless reported differently  Q25/50 compared to AT group  -Higher expression of superoxide dismutase, catalase and GSH peroxidase  -Higher expression of nuclear factor erythroid 2–related factor 2 and heme oxygenase 1  -Lower ICAM-1 levels (no difference in dose)  -Decreased STAT3 levels  Q50 compared to other groups  -Decreased expression of inflammatory markers NF-kB and IL-1B  Q25/50 compared to Diclofenac group  -No difference in downregulating MMP9 and MMP13 levels | NR |
| Kim et al.  (2022) (36)  Analyzing the efficacy of treatment | 9-week-old rat  (No amount reported)  Groups:  Control (c)  AT  CXB 1 injection/Week  CXB 3 Injection/week  PCNP 1mg only 1 injection  PCNP 4mg only 1 injection | After 4 weeks | Injection with collagenase type I 50uL | Injection with PPZ-CXB nanoparticle (PCNP): PCNP (CXB 1 mg) and PCNP (CXB 4 mg) | CXB compared to AT  -Higher expression of hydroxyproline  -More enhanced collagen regeneration  CXB3 compared to CXB 1  -More tendon regeneration | CXB compared to AT  -Higher expression of IL-4, IL-10  -Lower expression of COX-2, IL-1, IL-6, MMP-3, MMP-13, and TNF-α | CXB compared to AT  -Higher stiffness value  -Higher tensile strength |
| Jiang et al.  (2022) (41)  Analyzing the efficacy of treatment | Eight-week-old C57BL/6  male mice  (No amount reported)  Groups:  -Sham  -AT -AT+FR  -FR | After 4 weeks | Injection with collagenase type I 20uL | Injection with Friedelin (40 μM, 20 μL) | FR compared to AT  -Increased structural order of tendons, reduced inflammatory cells, better alignment collagen fibers, reduces neovascularization  -More high-strength produced collagen fibers | FR compared to AT  -Decreased expression of Dcn, Scx, Mkx and Tnmd  -Lower expression of F4/80+, Il-6, TNFa and IL1-B | FR compared to AT  -Increased failure load and ultimate stress |
| Xu et al.  (2022) (113)  Comparing the efficacy of treatment | 40 male 4-week-old Sprague-Dawley rats.  Groups:  -Control (n=4)  -Saline (n=12)  -AT (n=12)  - ASC-Ectos and ASC-Exos (n=12) | After 3 and 4 weeks | Injection with collagenase type I 25uL | Locally injected ASC-Ectos and ASC-Exos derived from extracellular vesicles derived from mesenchymal stem cells (: ASC-Ectos and ASC-Exos.  10^10^ exosomes suspended in 25 mL of saline in the left Achilles tendon and 10^10^ ectosomes suspended in 25 mL of saline in the right Achilles tendon. | ASC-Exos compare to ASC-Ectos  -Better histological score  -Less inflammation and spindle like cells  -Tighter fiber structure and less angiogenesis  ASC-Ectos compared to saline  -Better histological scores | ASC-Exos compare to ASC-Ectos  -Higher expression of collagen type 1 and CD206  ASC-Ectos and ASC-Exos compared to saline  -Decreased expression of collagen type 3 | ASC-Exos compare to ASC-Ectos  -Better ultimate tensile stress  ASC-Exos compare to saline  -Better failure load and ultimate tensile strength  ASC-Ectos compare to ASC-Exos  -Higher failure load, elastic modulus and ultimate tensile stress |
| Gundogdu et al. (2021) (111)  Analyzing the efficacy of treatment | 40 12–16-week-old Wistar albino male rats  Groups (n=10 per group):  -Control (C)  -Collagen+injury (Col)  -Docosahexaenoic(300mg)+injury (DHA300)  -Docosahexaenoic(100mg)+injury (DHA100) | After 8 weeks | Injection with collagenase type I 30uL | Oral gavaged with:  -Collagen 7.2 mg/kg/day  - Docosahexaenoic acid 300 mg/kg/day  - Docosahexaenoic acid 100 mg/kg/day  For 5day/week for 8 weeks | Col, DHA100 and DHA300 compared to control  -Inflammatory cells infiltration (macrophages and lymphocytes)  -Fibroblast and fibrocytes proliferation  -Less degeneration  DHA100 and DHA300 compared to Col and C  -Gaps between tendon fibers and calcifications  Col and DHA300 compared to C and DHA100  -Higher vascularization | C compared to Col, DHA100 and DHA300  -Decreased expression of TNF-B1, MMP-13 and IL-1B  Col, DHA100 and DHA300 compared to control  -Higher expression of collagen type I | Col, DHA100 and DHA300 compared to control  -Higher ultimate tensile force, yield force and stiffness |
| Piccionello et al.  (2021) (85)  Analyzing the efficacy of treatment | 32 tendons of 16 female sheep (Appenninica)  Groups:  -AAMG treatment (n=16)  -No treatment control (n=16) | After 8 weeks | Injection with 500 UI of type IA collagenase | Intratendinous Injection of adipo-autologous micro grafts (AAMG) 2.5 mL | Treatment relative to control group:  -Lower presence of necrosis, damaged fibers, inflammatory infiltrative process.  -Lower aspect of edema and myxoid | Treatment relative to control group:  -Higher expression of collagen type 1, FVIII (more active neo-angiogenesis)  Control relative to Treatment group:  -Overexpressed Collagen type 3 and TGF-ß1 in fibroblast and inflammatory cells | Mean Rupture force: Treatment group 28.3 N/mm^2^  and control 27.8N/mm^2^  Total rupture force: Treatment group 10.2 kN and control 9.87 kN |
| Nobre Evangelista  (2021) (62)  Analyzing the efficacy of treatment | 30 12-weeks old male Wistar rats  Groups:  -Control (n=8)  -Tendinitis (n=11)  -LED(n=11) | After 8 days | Injection with 100 uL of collagenase type I | Percutaneous application of Light-emitting diode  630 ± 20 nm, 300 mW, 9 J/cm^2^, 0.3 W/cm^2^ for 30s  After 1h for 7 days | Treatment compared to no treatment  -An increased expression of fibroblasts  -Increased neovascularization and collagen fiber alignment  -Decreased number of fat cells  -Reduced necrotic area | Treatment compared to no treatment and control  -Higher expression of HSP70 protein  Treatment compared to no treatment  -Increased amount of collagen | NR |
| Zhang et al.  (2020) (28)  Analyzing the efficacy of treatment | 24 3-months old female C57BL/6J mice  Group (n=6 per group):  - Cage activities (control)  - Daily metformin  - Intensive treadmill running  - Intensive treadmill running with metformin treatment | After 24 weeks | Intensive treadmill running 15m/min for 3h/d, 5d/wk for 24 weeks | Daily intraperitoneal injection of metformin (HMGB1 inhibitor) (50mg/kg) for 24 weeks | Group 4 relative to group 3:  -Decrease in frequency of round-shaped cells to levels similar to control samples  - Inhibited proteoglycan production  - Inhibited expression of chondrogenic markers | Group 4 relative to group 3:  -Blocked release of HMGB1  -Decreased CD68-positive and COX-2 cells, PGE_2_ production  - Inhibited ITR-induced Sox-9 and collagen type II expression | NR |
| Perucca Orfei et al.  (2020) (74)  Analyzing the efficacy of treatment | 68 12-week-old male Sprague Dawley rats  Groups PEMFs treatment:  4 Early acute phase (Day 1)  32 Mid-acute phase (Day 7)  6 Late acute phase (Day 15)  22 No PEMFs | After 15 up to 45 days | Injection with 3m mg/ml (185 IU/mg) collagenase type I | Percutaneous full-body exposure: Pulsed electromagnetic fields (PEMFs) 1.5 mT and 75Hz  8hr/day for 7 or 14 days | Treated relative to untreated Mid-acute Phase group:  -Restoration of physiological fiber structure and cell morphology  - Decrease of cellularity inflammatory infiltrates and vascularity  Late acute phase group:  -General improvement of tissue structure (Less evident than MAP group) | NR | NR |
| Jiang et al.  (2020) (41)  Comparison of treatment efficacy | 28 New Zealand White rabbits  Groups:  -Normal (n=4)  -Saline (n=8)  -Treatment (n=16) | After 3 or 6 weeks | Injection with collagenase type 1 110uL | Intratendinous injection of  - Lp-PRP 0.2mL (Leukocyte poor) in the left Achilles tendon  -Lr-PRP 0.2 mL (Leukocyte rich) in the right Achilles tendon | Lr-PRP compared to Lp-PRP and saline group:  -Better fiber structure and less angiogenesis in the Lr-PRP group compared to the Lp-PRP group  -More mature collagen fibers  Lp-PRP compared to saline:  -Lower histological scores overall  Treatment group compared to normal healthy tendons:  -Worse histological score | Lr-PRP compared to Lp-PRP and saline group  - Lower expression of collagen III  - An increased expression of collagen I VEGF and VEGF receptor (However, Decreased after 6 weeks in Lr-PRP)  - Higher expression of Arg 2 (decreased after 6 weeks), CD163 and IL-10  - Higher expression of CD163  Treatment group compared to saline:  - Lower levels of TNF-B | Lr-PRP compared to Lp-PRP and saline group:  - Higher failure load, stiffness, and tensile stress after 6 weeks |
| Li et al.  (2020) (56)  Comparison of early (after 1 week) or late (after 13 weeks) administration of treatment | 32 New Zealand White rabbits  Groups:  Control (n=6)  Saline-1 and PRP-1 (n=13)  Saline-2 and PRP-2 (n=13) | After 6 weeks | Injection with collagenase type I 300UI/rabbit, 260u/mg | Intratendinous injection with  -200-μl saline (Saline-1) in left Achilles tendon after 1 week  -200-μl autologous Lr-PRP (PRP-1) in the right Achilles tendon after 1 week.    After 4 weeks 13 other rabbits get the same treatments. (Saline-2 and PRP-2) | PRP-1 compared to Saline-1 and PRP-2  - Better healing results and histology score (Better fiber arrangement, structure, angiogenesis, rounding of nuclear, inflammation, and cell density)  - Increased CD163+ cells  - Thicker collagen fibrils compared to PRP-2 | PRP-1 compared to PRP-2  - Higher expression of Collagen type I in PRP-1 group  - Higher expression of collagen type III in PRP-2 group  - Higher expression of MMP-1 and MMP-3 in the PRP-2 group.  PRP compared to Saline  -Higher expression of IL-6 in PRP-2 compared with Saline-2 and PRP-1  -Higher expression of IL-10 in PRP-1 than saline-1 and PRP-2 group | -Failure load greater in the control group than Saline-1, Saline-2 and PRP-2 group. No difference between PRP-1 and PRP-2 group |
| Sánchez-Sánchez et al.  (2020) (57)  Analyzing the efficacy of treatment | 15 8-weeks old male Sprague-Dawley rats  Groups (n=5 per group):  -Healthy control (C)  -Tendinopathy control (T1)  -No treatment (T2)  -Percutaneous electrolysis (T2+PE)  -Sham needling (T2+N) | After 7,14 and 21 days | Injection with 250 UI collagenase type I 30uL | Percutaneous electrolysis with electrical current of 3 mA applied at 3 punctures 4s each. Three sessions, 1x/wk after 7,14 and 21 days | T2+PE compared to T2  -More signs of inflammation and presence of leukocytes  -Increase of tenocytes, loss in ECM organization, less ordered collagen fibers, and neovascularization similar between T2, T2+PE, and T2+N groups | T2+PE compared to T2  -Increase of COX2, MMP9, VEGF, MMP2, Collagen type I and III and Scx  T2+N compared to T2  -An increased expression of MMP2, MMP9 Collagen type I, and VEGF but lesser than T2+PE | NR |
| Rezvani et al.  (2019) (88)  Analyzing the efficacy of treatment | 493 12-week-old male mice (C 57B1/6)  Groups:  -Un-injured  - TGF  TGF-rHuPH20 | After 6h, 9 days, or 25 days after second rHuPH20 treatment | Injection with 2 doses rHuTGF 100nginto the calcaneal fat pad and bursa adjacent to Achilles tendon | Injection in retrocalcaneal bursa with Recombinant Human Hyaluronidase (rHuPH20) 10mg/ml after 24hr | Treatment group compared to TGFB1-induced group:  -After 9days reduced amount of glycosaminoglycans  -After 25 days both TGFB1-induced and TGFB1+Hyaluronisase groups showed chondroid removal and normal appearance of Achilles tendon and peritenon  -Rapid and extensive clearance of accumulated aggrecan/hyaluronan | Treatment group compared to no treatment:  -An increased expression of Ier3, Rel, Tlr2, Tnfrsf1b (Cell Fate), Adora2b,  Cdh1, Dcn, Has1, Wisp1 (ECM turnover), Pkm (energy metabolism), Ccl2, Ccl7, Cd80, Cxcl10, F10,  Infb1, Mif, Il12a, Ptx3 (inflammation) in the acute phase (6h after treatment injection)  -Decreased Ier3 | TGFβ and  TGFβ+Treatment group compared to uninjured  -Decreased maximum loads  TGFβ+treatment group compared to the TGFβ group.  - After 25d maximum  stress, stiffness, and modulus were more decreased |
| Kokubu et al.  (2019) (86)  Analyzing the efficacy of treatment | 36 6-week old male (C57BL/6J) mice  Groups (n=18 per group):  -ASC  -Control saline | After 9 days, 2 and 4 weeks | Injection with 1% type I collagenase (dissolved in 20 uL of phosphate-buffered saline (PBS) | Intratendinous injection of adipose-derived stem cells 1 x 10^7^ cells/mL in PBS (20uL) after 1 week | Treatment compared to no treatment  -Less severe degenerative changes at 2 and 4 weeks  -Improved alignment of collagen fibers  -Less ground substance deposition between collagen fibers  -A decreased area of calcification  -Ectopic mineralization is not seen in some tendons | Treatment compared to no treatment  -Both groups high cellularity after 9 days  -A decreased expression of IL-1B, Glut1 and CA9  -An increased expression of VEGF and CD31 | NR |
| Facon-Poroszewska et al. (2019) (75)  Analyzing the effect of combining treatments | 14 five-year-old sheep (Polish mountain sheep breed)  Groups:  -Control (N=1)  -PRP+Radial pressure wave (PRP+RPWT) (n=7)  -Adipose-derived stem cells + Radial shock wave group  (ADSCs+RPWT) (n=7)  The right limb of both treatment groups was the negative control for the use of RPWT | After 18 weeks | Injection with Collagenase type 1A-S 100IU in a volume of 0.2 ml | PRP+RPWT  Intratendinous injection of 0.4ml PRP on day 14  ADSCs+RPWT  Intratendinous injection of ADSCs on day 14.  Left limbs got additional percutaneous RPWT - 0.15 mJ/mm2, 8 Hz, 1000 impulses divided into the dorsal, medial, and lateral sides of the Achilles tendon area). The RPWT procedure was then repeated twice more at 7-day intervals | ADSCs+RPWT group compared to right limb  - The histological image was similar to healthy tendons.  - Fibroblasts shaped like mature tenocytes  - Smallest number of cells  RPWT+PRP compared to right limb  -RPWT+PRP showed an elevated rate of neovascularization. | ADSCs+RPWT group  - The largest amount of collagen type 1 arranged in a more parallel manner | NR |
| De Girolamo et al.  (2019) (89)  Analyzing the efficacy of treatment | 72 8–9-week-old Sprague Dawley rats  Groups:  -Control: Left Achilles tendon  (n=24 Healthy and n=48 sham(dry needling))  -Treatment: Right Achilles tendon | After 2 or 4 weeks | Injection with collagenase type I 3mg/mL 185 IU/mg | Intratendinous injection with 40 uL of either -Amniotic suspension Allograft (ASA) (collagenase + ASA group, n = 24),  -saline (collagenase + saline group,  n = 24), or  -untreated (collagenase group, n = 24) after 7 days | ASA group compared with the saline group  -Improvement in tissue structure, fiber organization, cell density, and fatty deposit formation  ASA group compared to healthy tendon  - Improved fiber alignment, as cell density and decreased presence  of inflammatory cells | - More abundant presence of residual human nuclei in comparison with collagenase-treated samples and sham group. | - Biomechanical testing at 28 days yielded a trend towards better results in the ASA group without  statistically significant differences between groups |
| Alquieri Fedato et al.  (2019) (60)  Comparison of single treatment effect and combination of treatments | 41 Wistar male rats  Groups:  -Stem cell (n=10)  -PRP (n=10)  -Stem cell+PRP (n=12)  - Eccentric exercise control (n=9) | After 4 weeks | Injection with collagenase type IA 250IU 10mg/ml | Intratendinous injection with:  -Stem cells out of 2ml blood  -Platelet-rich plasma out of 1-2 ml blood  -Both were carried out at the same time | - No significant differences in histological scores between groups according to morphology and density of tenocytes, presence  of hemorrhage, neovascularization, inflammatory cell infiltrate, linearity and undulation of collagen fibers, and epithelial thickness | Percentages of type 1 collagen in tissues:  SC group 61.82, PRP group 41.18, SC +PRP 49.29  ET group 40.09  Percentages of type III collagen in tissues:  SC group was 38.17, PRP group 58.81, SC+PRP group 50.70 ET group 59.90 | -No difference between groups in tensile and yield strength  -PRP Group had the best maximum deformation  -SC group had the best elastic modulus |
| Ahrberg et al.  (2018) (90)  Analyzing the efficacy of treatment | 6 horses (2-10 years old), 3 male and 3 female (Standardbred)  Groups:  -One hind and one forelimb injected with MSC  -Contralateral limb autologous serum control | After  0, 1, 2, 3, 4, 6, 8, 12 and 24 weeks | Injection with collagenase type I 0.4 mL 250 IU 4.8 mg/ml and minor mechanical tissue disruption in the superficial digital flexor tendon of all 4 extremities | Intratendinous injection with Mesenchymal stromal cells (MSC) (10^7^ Cells in 1 ml autologous serum) obtained from subcutaneous adipose tissue after 3 weeks | -After week 3 MSC group more frequent occurrence of macrophages and perivasculitis,  -MSC group had decreased percentage of healthy crimp and a higher percentage of erythrocytes in  suggesting increased inflammation and  vascularization.  -After 24 weeks lower cellularity in MSC groups but other parameters not different | MSC injection compared to contralateral  -No significant differences in collagen 1A2, collagen 3A1, decorin, tenascin-C, and scleraxis and osteopontin | NR |
| Bitterman et al.  (2018) (109)  Analyzing the efficacy of treatment | 307 12-weeks old Mature male mice (C57BI/6)  Groups:  -Control  -3 day  -9day  -IBU 3-25 days (E-IBU)  -IBU 9-25 days (L-IBU)  -IBU 3-25 days (UI-IBU) | After 3,9 and 25 days | Injection with active rhTGF-B1 (human recombinant) 100ng on day 0 and 2 | Oral administration of Ibuprofen (ibu) diluted with purified water to a concentration of 1mg/ml. Initiated after 3 or 9 days for a period of 22 or 16 days respectively. | Ibu compared to no ibu  -Elevated levels of chondroid  -Increased blood vessels in the adjacent fat pad  -Expression of multiple groups of GSI-positive cells  -Delayed clearance of pro-inflammatory matrix  -Prolonged EC< remodeling | Ibu compared to no ibu  -Delayed time to normalization of NFκb target and wound-healing genes  -A much higher expression of Cxcl5,  Col3a1, Il6, Mmp9, Col5a1, Cxcl3 and Ptgs2 genes  E-Ibu compared to L-IBU  -Higher expression of NFκb target and wound-healing genes | -IBU dosing resulted in a loss in stiffness and elastic modulus  - No statistically significant differences regarding the maximum load, maximum stress, displacement at maximum force, and strain at maximum stress between groups |
| Chen et al.  (2018) (87)  Comparison of single treatment effect and combination of treatments | 60 Sprague Dawley Rats  Groups:  -Physiological saline,  -rhPDGF-BB group  -hADSCs group  -rhPDGF-BB + hADSCs group | After 2 and 4 weeks | Injection of collagenase in the left hind leg  **Dose NR** | Intratendinous injection after 4 days with:  -Human adipose-derived stem cells (hADSCs) 1 x 10^5^ cells  -Recombinant human platelet-derived growth factor-BB  (rhPDGF-BB)10ng/ml  -Combination of both treatments | NR | Treatment compared to saline  -An increased expression of collagen type I, Scx, p-PI3K/Akt and TNC expression  -Downregulation of miR-363  -Stronger upregulated expression in combination treatment  Combination treatment  -rhPDGF-BB promoted the proliferation of hADSCs | -Contralateral healthy tendons strongest biomechanical properties  -All treatments improved biomechanical properties  -Combination treatment improved the stiffness (22.3 ± 1.3 N/mm) the stress (2.1 ± 0.2 MPa) and Maximum load to failure (47.4 ± 1.4 N) the best |
| Fjell Naterstad et al.  (2018) (64)  Comparison of treatment efficacy | 200 8/10-weeks old female Wistar rats  Groups (n=12 per group):  -Healthy control (control);  -Injury+no-treatment control (NT)  -Injury+LLLT (LLLT)  -Injury+diclofenac (NSAID)  -Injury+dexamethasone (GCS) | After 12, 24, 48, 72 hours  or  5, 7, 14, 21, 28, and 60 days. | Injection with collagenase 100 mg/ml | -Diclofenac 1.1mg/kg orally administered 2x/day for 7 days  -Percutaneous Low-level laser therapy (LLLT) 810 nm, with a power of 100mW in a mean spot size area of 2.8mm2 for 30s with 3J dose 1x/day for 7 days  -Dexamethasone 0.02mg/kg intramuscular injection 1x/day for 7 days | -NSAID and GCS treated group had decreased inflammatory cells during the first 2 days  -Dexamethason had a longer inflammatory phase and slower normalization of tendon quality compared to NSAID  -LLLT treated group had the best-improved hemorrhage, plasma leakage, neutrophil and lymphocyte count, and preserved tendon morphology  compared with the other groups. | -The relative amount of collagen I was significantly higher in the LLLT  group compared with the GCS group  -By birefringence analysis, NSAID and GCS delayed the reorganization of collagen fibers compared to LLLT. | NR |
| Gong et al.  (2018) (110)  Analyzing the efficacy of treatment | 60 Male Wistar rats  Groups (n=12 per group):  -Control  -Tendinopathy induced 7days  -Tendinopathy+Piperine (7days)  -Tendinopathy induced 21 days  -Tendinopathy+Piperine (21days) | After 7 or 21 days | Injection with 10mg/ml collagenase | Intragastrically administration of piperine 100mg/kg/day for 7 or 21 days | Piperine treatment in comparison with injury group  -Better morphological structure of the tendon  -Improved crimped and organized collagen fibers | Piperine treatment compared to injury group  -Similar levels of glycosaminoglycans as control group after decreased level in tendinopathy  -Improved values of hydroxyproline levels  -Reduced collagen type III levels (But mild elevation when compared to control group)  -Decreased levels of MMP-2 and MMP-9 and, p-ERK levels | NR |
| Da Ré Guerra et al.  (2017) (65)  Analyzing the efficacy of treatment | Male Wistar/Uni rats  Groups (n=5 per group):  -Control  -No treatment  -Treatment after 1 and 3 hr | After 7 hours | Intratendinous application of carrageenan | Percutaneous Low-level laser therapy with 830 nm, 40 mW, 4J/cm^2^ for 16 s after 1 and 3 hr | Treatment and no treatment group compared to control  -Higher cellularity and thicker epitenon | Treatment compared to control  -No difference in non-collagenous proteins  Treatment compared to no treatment  -No difference in MMP-2  -Increased MMP-9 expression  -Decreased non-collagenous protein expression | NR |
| Yan et al.  (2017) (80)  Comparison of efficacy of leukocyte concentration in PRP administration | 28 New Zealand white rabbits  Groups:  -Normal (N=4)  -Leukocyte poor-PRP (N=8)  -Leukocyte rich-PRP(N=8)  -Saline(N=8) | After 5 weeks | Injection with collagenase type 1 300UI/rabbit 260 u/mg | Intratendinous injection of Lp-PRP 0.2mL (Leukocyte poor)  Or  Lr-PRP 0.2 mL (Leukocyte rich) | Lp-PRP compared to Lr-PRP and saline group:  -Better total histological score  -Decreased inflammation score  -Better cell density  -Better collagen structure and arrangement  -More mature collagen fibers  -Tendency to form larger fibrils | Lr-PRP compared to Lp-PRP and saline group:  -An increased expression of IL-6  Lr-PRP compared to Lp-PRP  -No difference in MMP-1, MMP-3 levels, Higher expression of TIMP-1  Lp-PRP group compared to saline:  -Increased level of TIMP-1, collagen type I and collagen type I:III ratio  -No difference in collagen type III expression  PRP(total) compared to saline  -Lower levels of MMP-1 and MMP-3 | NR |
| Watts et al.  (2017) (94)  Analyzing the efficacy of treatment | 17 adult Quarter-Horse-type  horses (age range 2-7 years)  Groups:  -MicroRNA29a treatment (n=9)  -Placebo (n=8) | After 2,4 and 16 weeks | Injection of collagenase type I 1000U in superficial digital flexor tendon (SDFT) in forelimb | Intratendinous injection with 1.5 mL of 100nM miR29a After 1 week | Treatment compared to placebo  -Better histological scores regarding cell density, vascularity, linear fiber, and polarized collagen  -Less peritendinous reaction | -miR29a downregulated in induced tendinopathy models  Treatment compared to placebo  -Suppressed expression of collagen type 3 | NR |
| Hsieh et al.  (2017) (105)  Analyzing the efficacy of treatment | 32 16-20 weeks old Adult male New Zealand rabbits  Groups (n=8 per group):  -Collagenase and Percutaneous soft tissue release (PSTR)  -Collagenase+sham  -Vehicle only+PSTR  -Vehicle-only+sham | After 35 days | Injection with collagenase type I 10mg/ml | Percutaneous soft tissue release with a disposable 18G beauty cosmetic blunt-tip micro cannula needle 30 days after tendinopathy induction | Col-PSTR groups compared with the sham group  -Decreased cellularity  -Less inflammation and cell infiltration | Col-PSTR groups compared with the sham group  -Decreased Calcitonin gene-related peptide (CGRP-IR)  -Reduction of Substance P value (SP-IR) in the spinal cord (L5-S2) was found in bilateral superficial laminae | NR |
| De Farias Marques et al. (2016) (66)  Analyzing the efficacy of treatment | 42 female Wistar rats (Norvegicus Albinus) of which 36 aged (18 months old) and 6 adults (12 weeks old)  Groups:  -Tendinopathy aged (n=18)  -Tendinopathy+Treatment aged (n=18)  -Adult control (n=6) | After 7, 14, or 21 days | Injection with 1mg/ml collagenase | Percutaneous photo biomodulation therapy at the wave length of 830 nm (±10 nm), 50 mW power, laser  beam 0.028 cm2, energy density of 107 J/cm2, power density  1.8 W/cm2, and energy 3 J per point 12hr after injury 3 times a week until euthanasia | NR | Aged tendinopathy group compared to adult control  -Reduced collagen type I expression  Treatment group compared to injury  -Restored collagen type I levels  -Decreased levels of collagen type III  -Attenuated the MMP-3 and MMP-9 increases  - Increased VEGF levels (After decrease by tendinopathy) | NR |
| Pedrozo Vieira et al.  (2016) (108)  Comparison of treatment efficacy  (Myotendinous Junction) | Male 60-days old Wistar rats  Groups (N=6 per group):  -Control (C)  -Tendinitis (TE)  -Tend + Green tea (GT)  -Tend+ Glycine diet (GD)  -Tend + Glycine+Green tea (GTD) | After 7 days | Injection with collagenase type I 10 uL 10mg/mL) | Orally administration of:  -Green tea leaves of Camellia sinensis were used and 700 mg/kg/day was administered for 7 days  - Diet containing 5% glycine was provided for 7 days | Treatment compared to no treatment  --Better compaction of collagen fibers | GTD compared to TE  -A decreased expression of proMMP-9 and MMP-8  -Higher expression of collagen type I and Non-Collagenous Proteins  GTD compared to control  -Similar proMMP-9 value  GD compared to other groups  -Higher hydroxyproline dosage  -Highest IL-1B count | NR |
| Oshita et al.  (2016) (47)  Analyzing the efficacy of treatment | 16 male 12-weeks old F344/NS1c rats  Groups (n=8 per group):  -Adipose-derived stem cells treatment (ASC)  -Saline (PBS) group | After 4 or 12 weeks | Injection with 250 UI collagenase type I | Intratendinous injection with 50 mL saline containing 5 x 10^5^ ASCs after 1 week | ASC compared to PBS group  -Lower level of degenerative changes  -Decreased levels of disrupted collagen fibers, cellularity, and hypervascularity  -Less ground substance deposition between collagen fibers | ASC compared to PBS group  -Higher density of collagen fibers  -Increased levels of collagen type I  -Decreased levels of collagen type III | NR |
| Gonzalez et al.  (2016) (82)  Analyzing the efficacy of treatment | 30 New Zealand rabbits  Groups:  -Control (N=6)  -Saline (PBS)  Divided into 2-time groups (n=12 total)  -Treatment divided into 2-time groups (n=12 total) | After 4 or 12 weeks | Injection with 250 UI bacterial collagenase | Intratendinous injection of autologous leukocyte-reduced platelet-rich plasma (Lr-PRP) 0.2mL after 10 days | Treatment group compared to control after 4 weeks  -No differences in histology score  -PBS worst histology tendon injury score  Treatment group compared to control after 12 weeks  -No differences in ground substance scores  Treatment compared to PBS  -Better cell density at 4 weeks. However, this decreased after 12 weeks | Lr-PRP group compared to control  -Higher expression of collagen type 1 at 4 weeks. However, after 12 weeks no difference between groups  -No difference in collagen type 3 expression  -Higher expression of VEGF at 4 weeks. However, no difference at 12 weeks  Treatment group compared to control  -Higher expression of collagen type 3  -PBS group highest collagen type 3 expression at 12 weeks | NR |
| Pedrozo Vieira et al.  (2015) (26)  Analyzing the efficacy of treatment | 50 Adult 60-days old Wistar rats  Groups(n=10 per group):  -Control  -Tendinitis 7 days (TE-7)  -Tendinitis + Glycine 7 days(TEG-7)  -Tendinitis 21 days (TE-21)  -Tendinitis + Glycine 21 days(TEG-21) | After 8 and 22 days | Injection with collagenase type I 10 uL 10mg/ml | -Oral diet containing 5% glycine | TEG-7 compared to other groups  -Thicker epitenon | TEG-21 compared to inflamed and other treated groups  -Higher amount of hydroxyproline almost similar to control  -Lowest concentration of non-collagenous proteins  TEG-7 and TEG-21 compared to no treatment  -Higher total sulfated glycosaminoglycans  TEG-7 compared to TEG-21  -Greater concentration of non-collagenous proteins  -Increased MMP-2 expression | TEG-21 compared to inflamed and other treated groups  -Greater maximum load |
| Torres-Silva et al.  (2015) (67)  Comparison of treatment efficacy | 30 male Wistar rats  Groups (n=5 per group):  -Control saline (C)  -Nontreated tendinitis (NT)  -Tendinitis treated with sodium diclofenac (D)  -Tendinitis treated with laser 1J (L1J)  -Tendinitis treated with laser 3J (L3J)  - No treatment (TEN) | After 2 hours | Injection of collagenase 100ug in the right leg | - Sodium diclofenac (Voltaren injectable®; Novartis 2.5 mg/kg) injected intramuscularly  in the gluteus muscle after 30min  -Percutaneous Low-level laser therapy (LLLT) 660nm with the power of 100mW in a core spot size  area of 0.028 cm2 with doses of 1J or 3J after 1 hr | NR | L3J compared to Diclofenac and L1J group  -Significant inhibition of IL-6 gene expression  L3J compared to the Not treated group  -Reduction of TNF-B1 gene expression  L1J and diclofenac group compared to the Not treated group  -An increased expression of IL-10  -An increased expression of IL-6  L3J compared to control  -Higher level of COX-2 expression. But lower than the diclofenac, Not treated, and L1J group | NR |
| Dallaudiere et al.  (2015) (81)  Analyzing the efficacy of treatment | 22 immunocompetent male Sprague Dawley rats  Groups:  -PRP treatment (n=7)  -Physiological serum (n=7)  -No treatment (n=6) | After 3, 7, 13, 18, and 25 days | Injection of collagenase type 1 250 UI 30uL | Intratendinous injection of 0.1 ml PRP (Platelet concentration equal 3 times than the concentration measured in the blood) | NR | Treatment compared to serum and no treatment  No significant changes in EPO, TNF-B, GM-CSF. VEGF, IL-1a, IL-1b, MCP-1, IL-2, Il-4, IL-5, IL-6, IL-7, IL-10, IL-12p70, IL-13, IL-17a, IL-18, MCSF, MIP-3a, Rantes | NR |
| Calandruccio et al.  (2015) (83)  Comparison of treatment efficacy | 160 3-month-old Sprague-Dawley rats  Groups (n=32 per group):  -Control group  -Sham saline group  -Autologous blood group  -Platelet-rich plasma group  -Steroid group | After 3,7 and 14 days | Injection with collagenase type I-S 30uL | Intratendinous injection with:  - 0.2mg of  Methylprednisolone  -PRP 30 uL  -Autologous blood 30 uL  -Platelet-rich plasma 30 uL | -Autologous blood group had almost normal tendon alignment compared to control and saline  -Autologous blood group had better improvement and order in fiber alignment than saline and control groups | NR | Loads to failure mean  Control 57.3±14.3  Sham 51.9±14.6  Steroid 56.2±18.2  PRP 46.7±12.5  AB 60.1±15  -Only saline and PRP were significantly better when compared to no treatment |
| Kamineni et al.  (2015) (76)  Analyzing the efficacy of treatment | 12 8-weeks old female New Zealand white rabbits  Groups (n=6 per group):  -Control  -Treatment | After 6 weeks | Injection of collagenase type 1 10mg/ml | Percutaneous ultrasonic debridement performed with a Tenex ultrasonic probe for 30 seconds after 3 weeks | Treatment compared to control  -Higher histopathological score  -Heterogenous fiber orientation and less ordered  -Higher cellularity  Treatment compared to no treatment  -Lower histopathological score  -Lower cellularity | Treatment compared to no treatment  -Lower levels of collagenase type I and III  -Higher level of collagenase type X  Normal compared to treatment and no treatment  -The lowest expression of collagen type I, III, and X | NR |
| Dallaudiere et al.  (2015) (81)  Analyzing the effect of combining treatments | 20 immunocompetent male Sprague Dawley rats  Groups:  -Right paw: Bevacizumab+PRP (B-PRP)  -Left paw: PRP only (PRP) | After 6, 18 and 25 days | Injection with collagenase type I 250UI | Intratendinous injection after 3 days with:  -Bevacizumab 0,05ml  -Leukocyte Poor-PRP 0,1 ml  -Bevacizumab 0,05ml + 0,05 Leukocyte poor-PRP | B-PRP compared to PRP  -Less fibrillar disorganization and neovascularization  -Both groups had the absence of macrophages | NR | NR |
| Pedrozo Vieira et al.  (2015) (42)  Comparison of treatment efficacy | 42 Male 60-days old Wistar rats  Groups (N=6 per group):  -Control (C)  -Tendinitis 7 days (TE-7)  -Tend + Green tea 7 days (GT-7)  -Tend+ Glycine diet +Green tea 7 days (GTD-7)  -Tendinitis 21 days  -Tend + Green tea 21 days (GT-21)  -Tend + Glycine+Green tea 21 days (GTD-21) | After 7 and 21 days | Injection with collagenase type I 10 uL 10mg/ml | Oral administration of:  -Green tea leaves of Camellia sinensis were used and 700 mg/kg/day was administered for 7 or 21 days  - Diet containing 5% glycine was provided for 7 or 21 days | GTD-7, GT-21, and GTD-21 had similar birefringence similar to the control group  -TE-21 had the highest birefringence compared to other groups  -GTD-7 had thinner epitenon that TE group  -No difference in epitenon thickness after 21 days | GTD-21 compared to other tendinitis and treated groups  -The highest concentration of glycosaminoglycans, almost similar to the control group  GTD-21 compared to GT-21  -No difference in MMP-2 expression  GT-7 compared to TE-7  -Higher expression of MMP-2 | GT-21 compared to TE-21  -Better maximum load almost similar to control |
| Imai et al.  (2015) (30)  Analyzing the efficacy of treatment | 12 male New Zealand white rabbits  Groups:  -Right leg used for treatment  -Left leg used as control | After 48 days | Injection with collagenase 30 uL 10mg/ml in right leg | Percutaneous Augmented Soft tissue mobilization (ASTM) with 1.5 N/mm^2^ pressure for 3 minutes after 3 weeks. 6 treatments in total on different days | Treatment compared with control  -More aligned collagen fibers | Treatment compared with control  -Decreased level of collagen type III fibers | Treatment compared with control  -Lower storage modulus  -lower loss modulus  -No difference in loss tangent |
| Zhang et al.  (2014) (79)  Analyzing the efficacy of treatment | 29 female 10-weeks old mice (C57BL/6J)  Groups:  -Treadmill running (n=25)  -Healthy Control (n=4)  -Needle penetration  -No treatment Control (N=5)  -Treatment (N=40) | Ranged from 0 min up to 3.5hr | Treadmill running: 13 meters/min for 15 min per day for 1 week followed by one bout of exhaustive running at the same speed.  Needle penetration:  Penetration with 18G needle | Percutaneous cold treatment in a Koolit refrigerant gel (Cold Chain Technologies) maintained at 8 ± 2°C for 30 or 60 min followed with resting time | NR | Treadmill+treatment compared to control  -Cold treatments for 30 min after running decreased PGE2 levels by 33.8% and treatment for 60min reduced it by 50.8%  -Reduced COX-2 levels  Needle penetration+treatment compared to control  -Decrease of PGE2 levels in all treatment groups. The highest decrease in 2.5h rest after the treatment group | NR |
| Solchaga et al.  (2014) (52)  Comparison of treatment doses and efficacy of single treatments | 108 Male Sprague-Dawley rats  Groups(n=18 per group):  -control (C)  -Saline control (S)  -rhPDGF-BB 3ug (R3)  -rhPDGF-BB 10ug (R10)  -PRP  -Triamcinolone 300 ug (TCA) | Day 0 and 7 and 21 days after treatment | Injection with collagenase type IA 50uL 10mg /ml | Intratendinous injection after 7 days with:  -Recombinant Human Platelet-Derived Growth Factor-BB (rhPDGF-BB) 3ug  - 8.3 x 10^9^ – 1.5 x 10^12^ platelets/L PRP  -rhPDGF-BB 10ug  -Triamcinolone 300 ug | TCA compared to S  -Decreased cell proliferation and inflammation  R10 compared to S  -Increased cell proliferation  -After 21 days no differences between treatment groups | NR | -R3 better maximum load and ultimate stress compared to PRP, S, and TCA  -R10 better maximum load and stiffness Ultimate stress and modulus at day 21 compared to saline, PRP, and TCA  -R10 better maximum load and stiffness at day 21 than R3 |
| Lacitignola et al.  (2014) (48)  Analyzing the efficacy of treatment | 10 2-year-old female 2-years old sheep (Berganasca breed)  Groups (N=3 per group):  -3 weeks after treatment  -4 weeks after treatment  -6 weeks after treatment  The right leg is used for treatment, left leg used as the negative control | After 3, 4, or 6 weeks | Injection with collagenase type 1A from clostridium histolyticum 400 UI | Intratendinous injection with Bone marrow mesenchymal cells (BM-MSC_RPF_) 6 x 106 in fibrin glue in the left leg  The right Achilles tendons  received the same volume of fibrin glue without BM-MSC_RPF_ | Treatment compared to control tendons  -Good architecture of collagen type I fibers in the extracellular  matrix | Treatment compared to control tendons  -Higher expression of collagen type 1  -Higher expression of CD34+ cells | NR |
| Urdzikova et al.  (2014) (91)  Analyzing the efficacy of treatment | 81 adult rats  Groups:  -hMSCs group (n=41)  -Saline group (n=40) | After 2,4 or 6 weeks | Injection of 0.3 mg collagenase | -Intratendinous injection with 1 x 10^6^ mesenchymal stromal cells (hMSCs) cells in 100 μL saline after 3 days | Treatment compared to no treatment  -Lower cellularity and more spindle-shaped cells  -Decreased vascularity  -Better organization of collagen fibers  -Denser tissue matrix | Treatment compared to no treatment  -Higher expression of collagen type I and III  -No difference in aggrecan and versican expression | Treatment compared to no treatment  -No difference in mean peak force  -Increase in stiffness |
| Xavier et al.  (2014) (63)  Analyzing the efficacy of treatment | 30 male Wistar rats  Groups (n=5 per group):  7-day experimental groups  -Healthy control saline (c)  -Tendinitis (TEND-7)  -LED therapy group (LED-7)  14-day experimental groups  -Tendinitis group (TED-14)  -LED therapy group (LED-14)  -LED group therapy after 7 days |  | Injection with collagenase 100uL 1mg/mL | Percutaneous Low-level light-emitting diode therapy 880±10 nm 22 mW, spot irradiation time of 170 s, 7.5 J/cm2  after 12 h, with a 48-h interval between irradiations. | NR | LED compared to control  -An increased expression of IL-10, the highest increase in LED-7  LED compared to TEND and control groups  -Higher expression of collagen type I and III  -LED after 7 days lower expression of collagen type I after 14 days but higher than control | NR |
| Labat Marcos et al.  (2014) (68)  Comparison of treatment efficacy | 60 male Wistar rats  Groups (n=12 per group):  -Control(C)  -Tendinitis treated with sodium diclofenac (D)  -Tendinitis treated with laser 1J (L1J)  -Tendinitis treated with laser 3J (L3J)  - No treatment (TEN) | After 7 days | Injection with collagenase 100 mg/ml | - Sodium  diclofenac (Voltaren injectable®; Novartis 2.5 mg/kg) injected intramuscularly  in the gluteus muscle after 30min  -Percutaneous Low-level laser therapy (LLLT) 810 nm, with a power of 100mW in a mean spot size area of 2.8mm2. with doses of 1J or 3J after 1 hr | NR | D compared to TEN  -Decreased MMP-3 expression  -Increased MMP-9 and MMP-13 expression  L1J and L3J compared to TEN  -Decreased expression of MMP-13, MMP-9, and MMP-3  -Decreased MMP-3 and MMP-9 in L1J less important | Load levels:  Control 35N, TEN 20N, L1J 50N, D 35N, L3J 30N  Maximum force and stretch  -L1J recovered control properties, while L3J and D degraded the properties  Stiffness  -Improved stiffness in treated groups, worst in TEN group  -However, L3J group results close to TEN |
| Dallaudiere et al.  (2014) (84)  Analyzing the efficacy of treatment | 30 immunocompetent male Sprague Dawley rats  Groups:  -Tendinosis+ tendon injected with PRP (PRPT+)  -Tendinosis- tendon injected with PRP  -Physiological serum group (ST+) | After 6, 12, 13, 18, and 25 days | Injection of collagenase type 1 250 UI 30uL | Intratendinous injection of 0.1 ml PRP (Platelet concentration equal 3 times than the concentration measured in the blood) | PRPT+ group compared to ST+  -Less fibrillar disorganization and neovascularization in the ST+ group | -Absence of Macrophages in all groups | NR |
| Gunes et al.  (2014) (107)  Analyzing the efficacy of treatment | 32 male New Zealand white rabbits  Groups:  No surgery control(n=4)  Sham control(n=12)  RF-mt(n=12) | After 6 and 12 weeks after treatment | Injection with 1600ng of PGE1 1x/week for 4 weeks in Achilles tendon | -Surgical Radiofrequency-micro tenotomy device was used to perform micro tenotomies surgically one week after injection | RF-mt compared to no surgery control  -Increase in vascularity | RF-mt compared to no surgery control  -No difference in VEGF and collagen type 4  -No difference in fiber structure and fiber arrangement at 12 weeks | NR |
| Dallaudière et al.  (2013) (98)  Analyzing the efficacy of treatment | 40 6-week-old immunocompetent male Sprague Dawley rats  Groups:  -Bevacizumab group (Bev)  -Sham | After 6 and 13 days after injury induction | Injection with collagenase type I 250IU 30uL | Intratendinous injection with bevacizumab 0.1ml after 3 days | Bev compared to sham  -Better fibrillar organization and Less neovascularization at day 6  -Similar fibrillar disorganization and neovascularization at day 13 (Less than day 6 in both groups) | -Absence of macrophages in both groups | NR |
| Casalechi et al.  (2013) (69)  Analyzing the efficacy of treatment | 30 male Wistar rats  Groups (n=5 per group):  -Saline  -Laser  -Tendinopathy 7 days (TE-7)  -Tendinopathy 14 days (TE-14)  -Tendinopathy+laser 7 days (TL-7)  -Tendinopathy+laser 14 days(TL-14) | After 7 and 14 days | Injection with 0.1 mL of collagenase | Percutaneous Low-level laser therapy 780nm, 22 mW 107 mW/cm^2^ 1.54J applied for 70s for a period of 7 or 14 days | NR | TL-7 and TL-14 compared to saline  -Higher expression of IL-10 and VEGF  -Lower expression of MMP-1  -An increased expression of MMP-13 | NR |
| Bell et al.  (2013) (53)  Analyzing the efficacy of treatment | 83 12-weeks old male mice (C57B16)  -Uninjured controls  -cage activity controls  -acute response group (48h after injection)  -Treadmill 2 weeks  -Treadmill 4 weeks | After 48h ,2 and 4weeks | Injection with 100ng active TGF-B1 in 6uL BSA in saline | Uphill treadmill running at 32cm/s, for 20min/day. 5 days/week for 2 or 4 weeks | Treadmill 2 weeks compared to cage activity group  - Lower increase of cellularity 1.3-fold (vs 1.7-fold)  Treadmill 4 weeks compared to cage activity group  - Lower increase of cellularity 1.6-fold (vs 2.1-fold)  Treadmill 2 and 4 weeks compared to cage activity group  Treadmill exercise prevented groups of rounded cells, with enlarged and rounded nuclei, and with each cell surrounded by its organized pericellular matrix. | Treadmill 2 weeks compared to cage activity group  -A decreased expression of collagen type I and III  -An increased expression of collagen type II and Fn1  Treadmill 4 weeks compared to cage activity group  -Tenfold reduction of collagen type I and 200-fold reduction of collagen type III  -Reduction of collagen type II and Can  -Decreased Adamts5 and MMP-3 expression  -A decreased expression of aggrecan and ADAMTS5 | Treadmill compared to cage activity control group and treadmill 2 weeks group  -After 4 weeks, recovery in maximum load, stiffness, maximum stress, and tensile modulus |
| Tsai et al.  (2013) (71)  Analyzing the efficacy of treatment | 40 12-weeks old Sprague-Dawley rats  -Normal control (N=10)  -Sham control with RF (N=10), -Tendinopathy with RF (N=10)  -Tendinopathy (N=10) | After 1, 3, 5, and 7 days | Injection with collagenase type I 0.3 mg from clostridium histolyticum | The universal radiofrequency generator was used percutaneously on pulsed mode at 70 volts, 2Hz, for a duration of 30 milliseconds,  at 42∘C for 60 seconds. | NR | RF treated compared to tendinopathy  -Less expression of substance P and calcitonin gene-related peptide  -Fewer nerve fibers  -Reduction of nerve fiber diameter  -Improved tendon tension | RF treated compared to tendinopathy  -Improved stance phase  -Prolonged time of stance phase  -Step length toward to normal  -Improved intermediary toe spread |
| Cinar et al.  (2013) (31)  Analyzing the efficacy of treatment | 36 12-weeks old adult Sprague-Dawley rats  Groups:  -Tendinitis  -Tendinitis+extracorporeal shockwave (ESW)  -ESW only  -Sham | After 3 weeks | Injection with 10 uL of 3% carrageenan eight times during 7 day period | Extracorporeal shockwave ESW was  applied percutaneously at a rate of 500 impulses in 5 minutes at 2 bars (comparative to 0.09 mJ/mm2) after 1 week | Control group  -Best collagen fiber density compared with other groups  -No significant difference between groups in terms of number of capillaries and adhesion | NR | Tendinitis+ESW compared to control  -Decreased failure of tendon load |
| Shah et al.  (2013) (93)  Comparison of treatment doses | 75 Male Sprague Dawley rats  Groups (n=15 per group):  -Control  -Sham control  -1.02 ug rhPDGF-BB (Low)  -10.2 ug rhPDGF-BB (middle)  -102 ug rhPDGF-BB (High) | After 7 or 21 days | Injection with collagenase type IA 50uL 10mg/ml | Intratendinous injection with Recombinant Human Platelet-Derived Growth Factor-BB (rhPDGF-BB) in doses of:  -1.02 ug  -10.2 ug  -102 ug | Middle and High compared to sham  -Increase in proliferating cells at day 6  -Equal number of proliferative cells a day 21  All Treatment compared to sham  -No differences in the histopathological score at 7 and 21 days  -All groups had a decrease in inflammation score  -All groups had an increase in median collagen organization and collagen density scores | NR | Low, Middle, and high compared to sham  -Low group had the highest stiffness (12.4±2.2N)  -Middle group had the highest maximum load to failure (26.8±1.5N) |
| Labat Marcos et al.  (2012) (50)  Comparison of treatment efficacy | 30 male Wistar rats  Groups (n=6 per group):  -Control(C)  -Tendinitis treated with sodium diclofenac (D)  -Tendinitis treated with laser 1J (L1J)  -Tendinitis treated with laser 3J (L3J)  - No treatment (TEN) | After 7 days | Injection with collagenase 100 mg/ml | - Sodium diclofenac (Voltaren injectable®; Novartis 2.5 mg/kg) intramuscularly injected  in the gluteus muscle after 30min  -Percutaneous low-level laser therapy (LLLT) 810 nm, with a power of 100mW in a mean spot size area of 2.8mm2. and power density of 3.57 W/cm^2^ with doses of 1J or 3J after 1 hr | NR | L3J compared to TEN and D  -Decreased expression of COX-2 (similar to control group  D, L1J, and L3J group compared to TEN  -A decreased expression of PGE_2_  Laser group compared to TEN  -Decreased expression of TNF-B  -A decreased expression of MMP-9  -A decreased expression of MMP-13  L3J compared to TEN, L1J, and D  -A decreased expression of MMP-3 | -L3J group had a similar force as the control group  -L1J had a similar force and stretch as the control group  -Diclofenac showed a lower rupture point in comparison with the control |
| Don Yoo et al.  (2012) (114)  Analyzing the efficacy of treatment | 45 male 6-weeks-old Sprague-Dawley rats  Groups:  -Control (n=3)  -Tendinopathy (n=27)  -Tendinopathy+ESW(n=15) | Ranging from 1 to 33 days | Injection with collagenase type I 1.2mg/kg | Percutaneous extracorporeal shockwave therapy 4Hz, 0.085 mJ/mm^2^ 1000 impulses for 4 sessions | Treatment compared to no treatment  -Increased vascularity, fibroblast activity, collagen organization and lymphocyte and plasma cell infiltration on day 4 and 12 Disorganization of the fibers at day 12 and Less chronic inflammation signs | NR | Treatment compared to no treatment  -Increase in fibrillary diameter |
| Ng et al.  (2012) (29)  Comparison of single treatment effect and combination of treatments | 25 female 3-month-old Sprague-Dawley rats  Groups (n=5 per group):  -Running with laser (RL)  -Running with stretching (RS)  -Running with laser and stretch (RLS)  -Running no treatment (RNT)  -Control (C) | After 9 weeks | Bipedal downhill  running treadmill  20 m/min with a 20^o^ decline 1h/day, 7d/week for 8 weeks | - Percutaneous cutaneous  therapeutic laser 660 nm, 8.8 mW, 10 kHz for 50s 3.4J/cm^-2^  -Passive stretching 10 min after the exercise session (Maintained for 10s and repeated 20 times)  -Combination (stretching first) | NR | NR | -RL had the most improved tensile strength and stiffness compared to other running groups  -Combination or stretching has no effect in improving biomechanical properties  -All groups decreased biomechanical properties compared to control |
| Chen et al.  (2011) (40)  Comparison of tenocytes from multiple origins | 44 rabbits  Groups (n=14 per group):  -Control group  -Treatment A (ATT-A)  -Treatment B (ATT-B) | After 4 and 8 weeks after treatment | Injection with collagenase type I 30 uL | Intratendinous injection with:  -Autologous tenocytes therapy-A: Tenocytes harvested from patellar epitendineum tissue (ATT-A)  -Autologous tenocytes therapy-B: Tendon sample of 3x3mm^2^ harvested  (ATT-B) About 10^4^ autologous tenocytes  in 30 uL suspension | ATT-A&B groups compared to control  -Better histology scores regarding fiber structure, arrangement, rounding of nuclei and inflamed cells at 8 weeks  -Reduced angiogenesis | ATT-A&B groups compared to control  -An increased expression of collagen type I  -No difference in collagen type III expression  ATT-A compared to ATT-B  -A slightly lower expression of collagen type I | Ultimate failure load (at 8 weeks):  -Control 234.6 ± 52.4 N  -ATT-A 364.3 ± 92.6 N  -ATT-B 370 ± 40.9 N  -Normal 352.2 ± 27.6 N  Mean stiffness (at 8 weeks):  -Control 27.1 ± 12N/mm  -ATT-A 48.9 ± 17N/mm  -ATT-B 58.8 ±16.7N/mm |
| Pires et al.  (2011) (59)  Analyzing the efficacy of treatment | 42 male Wistar rats  Groups:  -Saline  -Laser  -Tendinopathy 7 days (TE-7)  -Tendinopathy 14 days (TE-14)  -Tendinopathy+laser 7 days (TL-7)  -Tendinopathy+laser 14 days(TL-14) | After 7 and 14 days | Injection with collagenase 0.1ml | Percutaneous Low-level laser therapy 780nm, 22 mW 7.7J/cm^2^ applied for 75s for a period of 7 or 14 days | NR | Treatment compared to no treatment  -Similar levels TNF-B and IL-B  -Decreased expression of IL-6, COX2, and TGF-B  -TNF-B only reduced at treatment at chronic phase (YL-14) | NR |
| Emrani et al.  (2011) (54)  Analyzing the efficacy of treatment | 48 nude rats (Crl:NIH-Foxn1^rnu^)  Groups:  Right Achilles tendon: Treatment  Left Achilles tendon: Sham | After 1, 3, 4, 15 days, and 1 month | Injection with collagenase 30uL 10mg/ml | Intratendinous injection with Human Umbilical Cord Perivascular cells (HUCPVCs) 2 x10^5^ cells in 30 μl of collagenase | Treatment compared to sham  -More linear collagen fiber arrangement at 30 days | - HUCPVC morphology of the  cells turned elongated coming from an ovoid form | Treatment compared to sham  **Tensile strength:**  Treatment: 5.39 ± 1.36 MPa  Control: 3.30 ± 1.27 MPa  -Increased young modulus in the treatment group |
| Labat Marcos et al.  (2011) (32)  Comparison of treatment efficacy | 150 male Wistar rats  Groups:  -Saline (S)  -Tendinitis treated with diclofenac (D)  -Tendinitis treated with laser 1J (L1J)  -Tendinitis treated with laser 3J (L3J)  -Tendinitis treated with laser 6J (L6J) | After 2 h up to 14 days | Injection with crude collagenase 30 uL | -Percutaneous Low-level laser therapy (LLLT) 810 nm, with a power of 100mW in a mean spot size area of 2.8mm2. and power density of 3.57 W/cm^2^ with doses of 1J , 3J, and 6J  -Diclofenac 1 mg/kg intraperitoneally injected in the abdomen | NR | -No differences in COX-1 expression between groups  -L3J group had better outcomes than the D group  L3J compared to other groups  -Highly decreased expression of COX-2 expression  -Decreased COX-2 derived PGE_2_ production  L1J compared to other groups  -Increased COX-1 derived PGE_2_ expression | NR |
| Martins et al.  (2011) (73)  Comparison of single treatment effect and combination of treatments | 50 adult male rats (Rattus norvegicus aged over 30 days  Groups:  -Treatment for 7 days  -Treatment for 14 days  Subgroups (N=10 per subgroup):  -Control;  -Ovis aries gel, topical use (massage);  -US + sterile lotion (oil-free);  -US + Ovis aries gel;  -Sterile lotion (oil free), topical use (massage). | After 7 or 14 days | Injection with collagenase 10 uL 10mg/ml | -Ovies Aries(sheep) lipid fraction gel 5% applied topically  -Percutaneous Ultrasound 10% pulsed mode, frequency of 1 MHz, 10% pulsed with an intensity of 0.5 W/cm2, oscillatory movements in 1 cm2 ERA for 2 minutes.  Daily treatment | -After 7 and 14 days Ovies Aries plus ultrasound group showed significant improvement in inflammatory cells | NR | NR |
| Xavier et al.  (2010) (58)  Analyzing the efficacy of treatment | 56 male Wistar rats  Groups (n=8 per group):  7-day experimental groups  -Control(C7)  -Tendinitis (TEND-7)  -LED therapy group (LED-7)  14-day experimental groups  -Control  -Tendinitis group (TED-14)  -LED therapy group (LED-14)  -LED group therapy after 7 days | After 7 or 14 days | Injection with 100ml of collagenase 1mg/mL | Percutaneous Low-level light-emitting diode therapy 880±10 nm 22 mW, the application time of 170 s, 7.5 J/cm2  after 12 h, with a 48-h interval between irradiations. | NR | LED compared to no treatment  -Decreased expression of IL-6, IL-1B , and TNF-B, however still higher than the control group. | NR |
| Ueda et al.  (2008) (99)  Analyzing the efficacy of treatment | 12 adult male New Zealand White Rabbits  Groups (n=3 per group):  Saline  Tendinitis+treatment  Tendinitis+saline  Control healthy | After 2 months | Injection with 1200 IU collagenase | Intratendon injection with Collagen oligopeptide 300 uL for 5 times wth interval of 3 days after 21 days | Treatment compared with saline  -Higher fiber density  -Increased Mass average diameter which is almost the same as a normal group  -Higher Glycosaminoglycan which consists of Hyaluronic acid and dermaran sulfate components | Treatment compared to control  -Decreased MMP-2 expression  Treatment compared to saline  -No significant difference in MMP-2 expression | NR |
| Naseri et al.  (2008) (72)  Analyzing the efficacy of treatment | 15 6-months old male New Zealand white rabbits  Groups:  -Left leg: Treatment  -Right leg: Sham | After 10, 20, and 30 days | Injection with collagenase 400IU 0.1 mL | Local injection with Bone marrow derived from the iliac crest and mixed with heparin (1000u/mL) | Treatment compared to no treatment  -30 days after treatment granulation tissue and inflammatory cell infiltration was greatly improved  -Better fiber alignment | NR | NR |
| Crovace et al.  (2008) (92)  Comparison of treatment efficacy | 6 2-year old sheep  Groups:  -cBMSC group (cBMSC)  -BMMNC group (BMMNC)  -Fibrin group (F)  -Saline solution group (S)  -Sham control (C) | After 8 weeks | Injection with 400 UI of Clostridium histolyticum Type 1A collagenase | -Intratendinous injection with Cultured bone marrow mesenchymal stem cells (cBMSC) 1.1 x  10^6^±1.4 x 10^5^ cells  suspended in a volume of 1 ml fibrin glue  -Bone marrow mononuclear cells (BMMNC) 101 x 10^6^ ± 26 x 10^6^ suspended in a volume of 1 ml fibrin glue | cBMSC- and BMMNCs group compared to control  -Newly formed collagen fibers  -Thin, well-oriented fibers with good cromp of pre-collagen mixed with well-differentiated and oriented red-stained fibers of mature collagen present | cBMSC- and BMMNCs group compared to control  -High expression of collagen type I  -A very low expression of collagen type I  -BMMNC-treated group had high expression of mononuclear cells which was partly CD34 stained | NR |
| Godbout et al.  (2006) (34)  Comparison of treatment efficacy | 180 8-weeks old male Wistar rats  Groups:  -Ambulation (A)  -Early exercise (EA)  -Tendinopathy+ambulation (TA)  -Tendinopathy+early exercise(TAE)  -Teninopathy+late exercise (TLE)  -Left tendon used as sham | After 3, 7, and 28 days | Injection with crude collagenase 30 uL 10mg/ml | **Early exercise:**  Voluntary exercise immediately after  injury on a wheel with counter  **Late exercise:**  Normal cage activity for 7 days followed by 21 days of exercise | TAE compared to sham  -Higher concentration of neutrophils at 7 days  -Hydroxyproline content returned to control values after 28 days | NR | -Early exercise did not strengthen tendon structure in longer-term  -Force at rupture point and ultimate stiffness tendency to be higher in sham tendons dan TAE at 28 days  -TLE had better Force at rupture points and ultimate stiffness outcomes |
| Chen et al.  (2004) (70)  Analyzing the efficacy of treatment | 123 3-month old Sprague Dawley rats  Groups (n=15 per group):  -Vehicle control  -Control ESW  -200 impulses  -500 impulses  -1000 impulses  The remaining rat was used for ESW for 200 impulses and killed at different time points | After 3 days, 1, 2, 4, 6, and 12 weeks after treatment | Injection with collagenase type I 250IU 30 uL | -Percutaneous Extracorporeal Shock Wave treatment 0.16 mJ/mm' energy-flux density, 1 Hz; for 200, 500, or 1000 impulses | 200 impulses compared to 500 and 100 group  -Better tendon fiber alignment and structure at 12 weeks  -Less loose fibrous tissue  200 impulses compared to sham  -Granulation tissue and inflamed cell infiltration improved at 4 weeks  -Increased vascularity and newly formed tendon tissue seen | 200 impulses compared to 500 and 100 group  -Increased DNA, glycosaminoglycan, and hydroxyproline content almost similar to control levels  -500 and 1000 impulses had lower levels  200 impulses compared to sham  -Increased PCNA expression  -Increase in cell proliferation  -An increased expression of TGF-B1 and IGF-1 at 1 and 4 weeks | 200 impulses compared to 500 and 100 group  -Better mechanical load to failure and stiffness  -500 and 1000 group decreased biomechanical properties |
| Tatari et al.  (2004) (103)  Analyzing the efficacy of treatment | 18 male Wistar white rats (11-16 months old)  Groups:  -Treatment control after 61 days  -Treatment control after 75 days  -Saline control after 61 days  -Saline control after 75 days | After 61 or 75 days | Injection with 0.1mL betamethasone sodium phosphate (Diprosporan) at 3-day intervals for 25 days | After 5 days of tendonitis induction. Local Injection with Hylan in the right limb in the 5-day interval until day 60 | Treatment compared to saline  -Decreased inflammatory cells  -Lower histopathological score in treated groups | NR | NR |
| Marsolais et al.  (2003) (33)  Analyzing the efficacy of treatment | Female Wistar rats  Groups:  Treatment (Diclofenac)  No treatment (Water  The contralateral tendon used as sham | After 1, 3, 7, 14, and 28 days | Injection with crude collagenase 30 uL | Administration of diclofenac dissolved in water at 1 mg/kg. (6 doses) (Gavaged) | Treatment compared to no treatment  -No difference in histological appearance after day 28  Treatment and sham compared to control  -Collagen fibers remained small and disorganized compared to control | Treatment compared to no treatment  -Reduced accumulation of PMN and ED1^+^ at day 1  -No effect on PMN and ED1^+^ in the core of tendon at day 28 | -Diclofenac treatment worsened biomechanical properties after collagenase injection |
| Yamamoto et al.  (2002) (101)  Comparison of treatment efficacy | 13 adult New Zealand white rabbits  Groups:  -Normal control (n=3)  -Hyaluronic Acid treatment (n=5)  -Beta-aminopropionitrile treatment(n=5)  Contralateral leg used as sham | 2 and 6 months after treatment | Injection with 400 IU collagenase | Local injection with:  -Hyaluronic Acid treatment (HA) 500 uL 10mg/ml  -Beta-aminopropionitrile treatment (BAPN) 100 uL 7mg/ml (5 sessions in 10 days intervals) | Treatment compared to no treatment  -Larger fiber diameters  -Similar glycosaminoglycan content  -Less irregular pattern  BAPN compared to HA  -Slightly smaller fibril diameter  -Less glycosaminoglycan  -Less normal collagen pattern  -Decrease of dermatan sulfate | NR | NR |
| Tatari et al.  (2001) (35)  Analyzing the efficacy of treatment | 28 female Wistar white rats  Group:  Left limb: Heparin  Right limb: Saline | After 60 days | Injection with betamethasone 0.1ml at 3 days interval for 25 days | Intratendinous injection with heparin 70U/kg | Treatment compared to no treatment  -More degenerative changes  -More loss of fibrillation  -More presence of capillaries and inflammatory cell infiltration  -Higher histopathological score | NR | NR |
| Gehlsen et al.  (1999) (77)  Comparison of treatment intensities | 30 30-weeks old white Sprague-Dawley rats  Groups (n=6):  -Tendinitis (A)  -Tendinitis plus light ASTM (B)  -Tendinitis plus medium ASTM (C)  -Tendinitis plus extreme ASTM (D),  -Sham | 1 week after the last treatment | Injection with collagenase 30 uL | **Percutaneous Augmented soft tissue mobilization (ASTM) every 4days (6 sessions)**  **Light pressure:**  **(0.5 N·mm^−2^)**  **Medium Pressure:**   1. N·mm−2   Extreme pressure  (1.5 N·mm^−2^) | C compared to B  -Increased number of fibroblasts  D compared to other groups  -The highest amount of fibroblasts  Groups B, C, and D compared to A  -Randomly arranged collagen fibers | NR | NR |
| Davidson et al.  (1997) (78)  Analyzing the efficacy of treatment | 20 Male 27-weeks old Sprague-Dawley rats  Groups (n=5 per group):  -Control(A)  -Tendinitis (B)  -Tendinitis plus ASTM (C)  -ASTM alone (D) | 10 days after last treatment | Injection with collagenase 30 uL | **Percutaneous Augmented soft tissue mobilization (ASTM) for 3 min on postoperative days 21, 25, 29, and 33** | C compared to B  -Similar misaligned collagen fibers  -Increased number of fibroblasts | -In all groups, the amount of collagen type I and III appeared to be similar  C compared to other groups  -More diffuse foci of collagen type III staining | NR |
| Williams et al.  (1986) (100)  Analyzing the efficacy of treatment | 16 4 -years old New Zealand white rabbits  Groups (n=8 per group):  -Treatment  -No treatment | After 1 month | Injection with bacterial collagenase 10mg/ml 0.05ml | **Intratendinous injection with10 units of heparin in 0.5ml phosphate-buffered**  **saline** | Treatment compared to no treatment  -More organized collagen alignment  -Less cellularity and vessel density | Treatment compared to no treatment  -Similar collagen concentration | NR |
|  |  |  |  |  |  |  |  |

# Appendix E – In vitro combined with in vivo animal studies

| Author & Year  Study aim  Reference | No. of Included animals & in vitro model  Animal breed  Assigned groups | Time of Analysis | Method of inducting AT | Treatment | Histological outcome | Biochemical outcome | Biomechanical outcome |
| --- | --- | --- | --- | --- | --- | --- | --- |
| Xu et al.  (2023) (95)  Comparing the efficacy of treatment | In vitro  Rat tendons digested with 3mg/mL collagenase I solution  In vivo  40 adult male Sprague-Dawley rats.  Groups (n=10/group)  -Sham  -Control(AT)  -Votalin  -CeO_2_ | After 14 days | In vitro  Tenocytes treaded with different concentrations of CeO_2_ solution for 24hr  In vivo  Injection with collagenase type I 50uL | In vitro  CeO_2_ NP cells added to medium  In vivo  Injection with 25uL/mL CeO_2_ NP (Nano particles) twice a week for 2 weeks  Administration of 1mg Votalin emugel | In vivo  Votalin compared to Control group  More regular and compact alignment of collagen fibers  Votalin and CeO_2_ compared to Control group  Prevented disruption and disorganization of collagen fibers | In vitro  -CeO_2_ alleviated Il-6 and Il-1B inflammatory response in tenocytes  -CeO_2_ decreased ROS changes in tenocytes  In vivo  Votalin and CeO_2_ compared to Control group  Higher expression of collagen type I  CeO_2_ compared to Control group  More enriched NRF2 in nuclei of the tenocytes | NR |
| Jiao et al.  (2023) (102)  Analyzing the efficacy of treatment | In vitro  Achilles tendon of one week old rats  In vivo  Eight week old male Sprague-Dawley rats.  Groups (n not mentioned):  -Control  -Tendinopathy  -Fullerenol 0.5 mg/group  - Fullerenol 1 mg/group | After 21 days | In vitro  Tendon cells were treated with 50ug/mL IL-1B  In vivo  Injection with collagenase type I 50uL | In vitro  Aqueous fullerenol  In vivo  Injection with 50 uL aqueous fullerenol with the concentration of 0.5 mg/mL and 1 mg/mL | In vitro  NR  In vivo  Fullerenol compared to AT  -Alleviation of impaired tendon fibers | In vitro  Fullerenol compared to AT tendon cells  -Higher expression of collagen type I and TNC  -Lower expression of MMP-3 and MMP-13  -Lower levels of ROS  In vivo  Fullerenol compared to AT  -Higher expression of collagen type I | NR |
| Wu et al.  (2022) (46)  Analyzing the efficacy of treatment | In vitro  Achilles tendon tenocytes of Sprague-Dawley rats.  Groups:  -Control  -RSL3 group  -RSL3+Farrerol  -Farrerol  Groups  In vivo  24 male Sprague-Dawley rats.  Groups (n=6/group):  -Control  -Tendinopathy  -AT+Farrerol  -Farrerol | After 4 weeks | In vitro  Tendon cells were treated with RSL3  In vivo  Injection with collagenase type I 30uL | In vitro  Farrerol  In vivo  Injection with 2 μg/100 μl of Farrerol | In vitro  In vivo  AT+Farrerol compared to tendinopathy group  -Less low stretch stress fibers  -Higher expression of TNMD | In vitro  RSL3+Farrerol group compared to RSL3 group  -Inhibited expression of FTL and FTH  -Increased expression of TfR1, GPX4 and SLC7A11  In vivo  AT+Farrerol compared to tendinopathy group  -Higher expression of tnmd, scx and mkx  -Less F4/80-positive cells infiltration  -Lower mRNA levels of the pro-inflammatory cytokines *Mcp1*, *Pge2*, *Tnfa*, *Il*-*1b*, *Il*-*6* and *Il*-*17* | AT+Farrerol compared to tendinopathy group  -Higher Young’s modulus and maximum stress |
| Micheli et al.  (2022) (96)  Comparing the efficacy of treatment | In vitro  Achilles tendon tenocytes of Sprague-Dawley rats.  Groups:  -Control  -FGF-2  -Fibroin  In vivo  24 male Sprague-Dawley rats.  Groups (n=6/group):  -Control  -Tendinopathy (AT)  -Tendinopathy+Fibroin  -Tendinopathy+triamcinolone | After 4 weeks | In vitro  Tendon cells were treated with FGF-2  In vivo  Injection with collagenase type I 20uL or carrageenan 20uL 0.8% | In vitro  tenocytes were incubated with fibroin at various concentrations (0.5 – 500 μg/mL).  In vivo  Fibroin (100 μg, 1 and 5 mg in 20 μL) was alternatively injected by a single intra-tendon treatment (day 2 after damage) or by peri-tendon injections on days 1, 3, 5, and 7.  Triamcinolone | In vivo  -Peri-tendon injection of fibroin 5 mg improved collagenase-induced damage,  -The intra-tendinous injections of fibroin were able to restore carrageenan-induced degenerative changes in the animals’ tendons  -A complete repair was achieved following triamcinolone treatment, collagen fibers appeared wavy, compact, and parallel arranged. | In vitro  AT+Fibroin compared to tendinopathy group  -Higher expression of decorin, scleraxin, TNC and tenomodulin  -Lower expression of FGF-2 | NR |
| Yamaura et al.  (2022) (97)  Analyzing the efficacy of treatment | In vitro  Tenocytes of 8 eight week old female Sprague-Dawley rats.  Groups:  -Without NMN and regular glucose  -NMN and regular glucose  -Without NMN and high glucose  -NMN with high glucose  In vivo  8 eight week old female Sprague-Dawley rats.  Groups (n=4/group):  -Control  -NMN | After 4 weeks | In vitro  Incubation of regular glucose (RG) group, and high-glucose (HG)  group.  In vivo  Injection with collagenase type I 30uL | In vitro  Nicotinamide mononucleotide (NMN) dissolved in saline  In vivo  Daily injections for 2 weeks of 1mL of NMN solution equivalent to  100mg/kg of NMN intraperitoneal | NR | In vitro  NMN+-HG compared to no NMN+HG  -Lower expression of NOX1, NOX4 and IL6  -Higher expression of SIRT1 and SIRT6  In vivo  NMN compared to control group  -Lower expression of NOX1 and NOX4  -Lower expression of IL-6  -Higher expression of SIRT1 and SIRT6 | NR |
| Ko et al.  (2022) (44)  Analyzing the efficacy of treatment | In vitro  Human tenocytes  -LPS -SPI  In vivo  28 eight week old female Sprague-Dawley rats.  Groups:  -Control (c) (n=4)  -Tendinopathy (AT) (n=12)  -Substance P Inhibtor (SPI) (n=12) | After 3 and 5 weeks | In vitro  Treated with lipopolysaccharide (LPS)  In vivo  Injection with collagenase type I 20uL | In vitro  Incubation with Substance P inhibitor  In vivo  Injection with Substance P inhibitor 20uL | In vivo  SPI compared to AT group  -Lower collagen disruption  -Lower proteoglycans and glycosaminoglycan’s deposition | In vitro  SPI compared to LPS(AT)  -Lower expression of COX-2, IL-6 and SCX  In vivo  SPI compared to AT group  -Decreased expression of IL-6 and NK1R | In vivo  SPI compared to AT group  -Higher tensile strength |
| Min Lee et al.  (2021) (15)  Comparison of treatment doses | In vitro:  Human tenocytes cultured on pre-coated flasks with collagen type 1  In vivo:  21 5-weeks old male Sprague-Dawley rats  Groups:  -Negative control(n=3)  -Positive control(n=6)  -High mitochondria(n=6)  -Low mitochondria(n=6) | After 2 weeks | In vitro:  Administration of TNF-B (10 ng/ML) for 24 hours  In vivo:  Injection with 0.6mg/ 40uL collagenase in both limbs | In vitro:  Administration of 1, 5, or 25 ug isolated mitochondria from Umbilical cord-mesenchymal stem cells  In vivo:  Intratendinous injection of 10ug(low) or 50ug(high) isolated mitochondria from L6 mice myoblast in 20 uL volume after 2 weeks | NR | In vitro:  TNF-1B compared with non TNF-1Bexposure:  -Enhanced TNMD and collagen type 1 expression and decreased MMP1 expression  -Decreased intracellular oxidative stress (ROS levels)  -Restoration of ATP content  - Inhibits TNF-B1, induced apoptosis (Decreased expression of BID, Bax, and increase of Bcl-2)  -Treatment suppressed IL-1B, and IL-6  In vivo:  Treatment compared to non-treatment  -Protein levels of Tenascin C and MMP1 decreased  -Increased collagen and MFN2 production  -Decreased BID, Bcl-2, TNF-B, Il-1B, Fis1, Drp1 and IL-6 levels | NR |
| Ruan et al.  (2021) (17)  Comparison of single treatment effect and combination of treatments | In vitro:  Cultured Tendon stem/Progenitor cells from rabbits Achilles tendon  In vivo:  33 New-Zealand White rabbits  Groups  -Normal control (NOR)  -Normal saline (NS)  -Triamcinolone acetonide (TA)  -PRP treatment  -TA+PRP treatment group | After 8 weeks | In vitro:  Cells treated with IL-1B  In vivo:  Injection with collagenase type I 300 UI 260U/mg | In vitro:  treated with 1ng/ml IL-1B, TA , 0.1 mg/mL and PRP or IL-1B and PRP  In vivo:  Peritendinous injection with Leukocyte-poor PRP  Or  Triamcinolone acetonide in combination with Leukocyte-poor PRP 200 uL after 2 and 3 weeks | In vitro  PRP and TA+PRP compared to no PRP  -Better characteristics such as the elongated spindle-shaped morphology of TSPCs  In vivo  PRP compared to TA+PRP  -More vascular infiltration, higher cell density, more small disordered collagen fibers  -More Col3 and immature Col1  -Less infiltration of inflammatory cells  -TA+PRP histological score compared to normal group | In vitro  PRP compared to no PRP  -Increased CHI3L1, MMP1, and MMP12  -Increased TNFRSF1B and HMOX1 (anti-apoptotic)  -Upregulated S100A12, IL1A, IL1B, and IL7  In vivo  TA+PRP compared to PRP  -High Col1 and low Col3 levels | TA+PRP compared to NS:  Maximum tension load greater in the treatment group  No difference with PRP group |
| Choi et al.  (2020) (20)  Comparison of single treatment effect and combination of treatments | In vitro:  Tenocytes obtained from the Achilles tendon of the rat  In vivo:  28 8-weeks old male Sprague-Dawley rats  Groups(n=4 per group):  -Control  -Collagenase  -Collagenase+ PLGA NPs  -Collagenase+ Hep-PLGA NPs  -Collagenase+ LF(1ug)/ Hep-PLGA NPs  -Collagenase+ LF(10ug)/ Hep-PLGA NPs  -Collagenase + LF solution (500ng) | After 4 weeks | In vitro:  Treated with IL-1B 100 ug/mL for 1 or 3 days.  In vivo:  Injection with collagenase 50 uL 50mg/mL in right hind limb | -Lactoferrin (LF) immobilized polymeric nano delivery system using poly  (lactic-co-glycolic acid) (PLGA) nanoparticles (NP) **LF PLGA NP 10mg**  -Polymeric nano delivery system using poly  (lactic-co-glycolic acid) (PLGA) nanoparticles (NP) **PLGA NP 10mg**  -Heparin modified  PLGA NP **Hep-PLGA NP**  **-** LF-entrapped heparin-  PLGA **LF/Hep-**  **PLGA NPs (1ug and 10ug)**  -Lactoferrin solution 500 ng | In vivo  -**PLGA and Hep-PLGA groups** still had severe disruption of collagen fibril organization  - **LF treatment and**  **both LF/Hep-PLGA/NPs treatment** prevented disruption of collagen fibrils  - **LF(10ug)/ Hep-PLGA NPs** most effective at preventing disorganization of collagen fibers  -- **LF treatment and**  **both LF/Hep-PLGA/NPs treatment** increased collagen type I, DCN, and TNC levels in comparison with other treatment groups | In vitro  - LF and the two types of LF/Hep-  PLGA NPs had prominent anti-inflammatory effects as reflected by their suppression of mRNA levels of pro-inflammatory mediators  - LF/Hep-  PLGA NPs demonstrated weaker anti-inflammatory effects than LF only.  In vivo  -LF and the two types of LF (1 μg  or 10 μg)/Hep-PLGA NPs markedly decreased mRNA levels of pro-inflammatory factors and proteases.  - LF(10 μg)/Hep-PLGA  NPs were very effective at suppressing mRNA levels of all pro-inflammatory  mediators. | -**LF- and LF/Hep-PLGA-treated groups** demonstrated greatly increased stiffness and tensile strength compared to other treatment groups and is almost at the level of the control group. |
| Liu et al.  (2020) (16)  Analyzing the efficacy of treatment | In vitro  Tenocytes obtained from 8-weeks-old mice  In vivo  12-week old mice(C57BL/6)  Groups:  -Control (n=6)  -H_2_O_2_-induced  -H_2_O_2_induces+treatment | After 3 weeks | In vitro  Treated with 500 uM H_2_O_2_ for 24 hr  In vivo  H_2_O_2_ (25 μl of 500 μM) over Achilles tendon 3x/week for 3 weeks | In vitro  Tenocytes were  pre-treated with Alda-1  In vivo  Subcutaneous injection Pre-administered with Alda-1 (20mg/kg) for six hours and subsequently injected with H_2_O_2_ | In vivo  Treatment compared with no treatment  -Inhibited apoptotic cell death of tenocytes  -Induced hypercellularity and neovascularization | In vitro  Treatment compared to no treatment  -Decreased level of caspase-3, caspase-9, cytochrome c, and Bax  -Decreased level of Grp78, IRE1α, PERK, CHOP, or active caspase-12 protein  -Blocked IL-1β and TNF-α upregulation  -Blocked down-regulation of collagen type 1 | NR |
| Choi et al.  (2020) (19)  Comparison of single treatment effect and combination of treatments | In vitro  Cultured tenocytes  In vivo  24 Male 8-weeks old Sprague-Dawley rats  groups (n=4 per group):  - Normal control  -Induced-Tendinitis  -Induced tendinitis + PMS  -Induced tendinitis + DEX (1%)/PMSs,  -Induced tendinitis + DEX (5%)/PMSs  -Induced tendinitis + DEX (10%)/PMSs. | After 4 weeks | In vitro  Il-1B(100 ng/mL) added to tenocytes  In vivo  Collagenase Type I 50 *μ*L administered to right Achilles tendon | Dexamethasone-containing porous microspheres (DEX/PMSs)  In vitro  Addition of  1.2 mg for DEX (1%)/PMSs  6.95 mg for DEX (5%)/PMSs  13.8 mg for  DEX (10%)/PMSs  In vivo  At 1 week, the rats were  Intratendinous injected with 50 *μ*L of carboxymethyl cellulose (CMC) containing  each microsphere (10 mg) with:  -60 *μ*g/rat for DEX (1%)/PMSs,  -347.5 *μ*g/rat for DEX (5%)/PMSs  -690 *μ*g/rat for DEX  (10%)/PMSs, | In vivo  DEX/PMSs compared to PMS alone  -Decreased collagen fiber breakdown  -DEX(10%)/PMS displayed the best therapeutic effect | In vitro  DEX/PMSs compared to PMS alone  -Decreases the level of COX-2, IL-  1β, IL-6, and TNF-α  -DEX(10%)/PMSs showed slightly lower levels than other DEX/PMSs groups  In vivo  DEX/PMSs compared to PMS alone  -Decreases the level of COX-2, IL-  1β, IL-6, and TNF-α  -DEX(10%)/PMSs showed a slightly lower best reduction compared to other DEX/PMSs groups | -Tensile strength and stiffness increased dose-dependently in the DEX/PMSs treated groups  -The tendinitis and PMS treated groups had much lower values compared to the control group |
| Wang et al.  (2020) (21)  Analyzing the efficacy of treatment | In vitro:  Tendon stem cells derived from rats cultured in Dulbecco’s  modified Eagle’s medium  In vivo:  24 male Sprague-Dawley rats  Groups (n=8 per group):  -Control  -Tendinopathy  -Tendinopathy+Aspirin | In vitro  3, 7, and 14 days.  In vivo  After 5 weeks | In vivo:  Injection with collagenase type I 30 uL (10mg/ml) | In vitro:  Treated with concentration of 0, 0.25, 0.5, 1, or 2mM aspirin for 24 hr or with 2mM aspirin for 3, 7, and 14 days  In vivo:  Intratendinous injection with Aspirin after 1 week for a period of 4 wk | In vivo:  Treatment compared to no treatment  -Better arrangement of collagen fibers | In vitro:  -An increased expression of GDF6, GDF7, and GDF11in the aspirin medium group compared to  induction medium group  -Aspirin promotes tenogenic differentiation of tendon stem cells  In vivo:  Treatment compared to no treatment  -Higher expression of TNC, TNMD, and SCX | In vivo:  Treatment compared to no treatment  -Better ultimate stress and young modulus |
| Zhao et al.  (2019) (22)  Analyzing the efficacy of treatment | In vitro  Tendon cells obtained from patellar and Achilles tendons of 3-4 months old female Sprague Dawley rats  In vivo  48 3-months old female mice  Groups(n=12 per group) divided in treatment and no treatment each:  -Control  -Moderate treadmill (MTR)  -Intensive treadmill (ITR)  -One-time treadmill (OTR) | After 3 and/or 24 weeks | In vitro  Tenocytes cultured in 3mg.ml collagenase type I and 10 μg/ml HMGB1 added  In vivo  Running speed 15 meters/min  -**MTR** 50 min/day, 5x/week  -**ITR** 3hr/day, 5x/week  -**OTR** More than 5hr until fatigue | In vitro  Administration of 200 μM Glycyrrhizin  In vivo  -Daily intraperitoneal glycyrrhizin (HMGB1-inhibitor) injection  -Injection with glycyrrhizin 15 min before treadmill running | In vivo  Treatment compared to no treatment  - Lt-ITR mice treated  with a daily injection of GL, no change in cell shape was observed  - Glycosaminoglycans accumulation  was prevented by GL administration in the group or ITR mice | In vitro  Treatment compared to no treatment  -Glycyrrhizin induced PGE2 and MMP-3 production  In vivo  Treatment compared to no treatment  -Daily Glycyrrhizin injection before ITR blocked the increase in PGE2 and MMP-3 production  -Glycyrrhizin inhibited the expression of SOX-9 and Col II | NR |
| Hoon Lee et al.  (2019) (43)  Comparison of administration type of treatment | In vitro:  Tenocytes isolated from Achilles tendon tissues of rats  In vivo:  New Zealand White Rabbits  Groups:  -Control (no treatment),  -Collagenase (collagenase injection),  -Tendinopathy and PCL  - Tendinopathy and diclofenac (5 mg) solution,  - Tendinopathy and DFN (1 mg)/PCL  - Tendinopathy and DFN (5 mg)/PCL | In vitro  3, 7, and 14 days.  In vivo  After 4 weeks | In vitro:  Administration of lipopolysaccharides of E.coli  In vivo:  Injection with collagenase type I 50 uL (50mg/ml) | In vitro:  Administration of diclofenac 500 ug/mL  In vivo:  Surgical placement of 1 and 5mg diclofenac-immobilized polycaprolactone (DFN/PCL) fibrous sheets (3x2cm)  **the positive drug control** group, diclofenac (5 mg) injected into the tendopathic Achilles tendon. | In vivo:  DFN/PCL sheets compared to PCL and diclofenac group  -Decreased number of inflammatory cells  -Restored collagen fiber arrangement  -DFN (5mg)/PCL showed the best decrease of inflammatory cells and overall restoration of collagen organization | In vitro:  Treatment compared to PCL group  -A decreased expression of inflammatory cytokines  DFN/PCL sheets compared to PCL group  -DFN/PCL less anti-inflammatory effect  In vivo:  Treatment compared to PCL group  -Diclofenac and DFN/PCL sheets were comparably and dose-dependently effective in decreasing the expression of inflammatory cytokines  DFN/PCL sheets compared to PCL group  -Higher expression of IL-4, IL-10, and IL-13 | Treatment compared to PCL and no treatment  -Better stiffness and tensile strength of the tendon tissues  - DFN (5mg)/PCL had the best increase in stiffness and tensile strength |
| Jeong et al.  (2018) (23)  Comparison of administration type of treatment | In vitro:  Tenocytes isolated from 8-week old Sprague Dawley(SD) Rats  In vivo:  24 male 8-week-old SD rats  Groups (n= 4 per group):  -Control (no treatment),  -Col (I) (collagenase treatment),  -Col (I) + PMSs,  -Col (I) + simvastatin,  -Col (I) + SIM (1 mM)/PMSs  -Col (I) + SIM (5 mM)/PMSs. | In vitro  After 3 days  In vivo  After 7 weeks | In vitro:  Treated with lipopolysaccharides  In vivo:  Injection with collagenase type I 50 uL (50mg/ml) | In vitro:  Simvastatin-loaded  porous PLGA microspheres (SIM/PMSs) added to tenocytes  In vivo:  Intratendinous injection with:  -SIM (1 mM)/PMSs 2.39 mg/rat  -SIM (5 mM)/PMSs 12.18 mg/rat  -Simvastatin (105 mg/rat) | In vivo:  SIM/PMS compared to PMS and no treatment  -Suppressed collagen matrix disruption  SIM/PMS compared to simvastatin  -More aligned collagen fiber organization  -Prevented collagen disruption | In vitro:  SIM/PMS compared to PMS and no treatment  -Decreased levels of MMP-3, COX 2, IL-6, and TNF-B  In vivo:  SIM/PMS compared to simvastatin  -Higher expression of hydroxyproline almost at the level of control  -Decreased expression of MMP-3, COX-2, IL-6, TNF-B and MMP-13 after 6 weeks  -The highest expression of IL-4, IL-10, and IL-13 after 6 weeks | In vivo:  Simvastatin compared to PMS  -Better stiffness and tensile strength  SIM/PMS compared to simvastatin  -Better stiffness and tensile strength |
| Kim et al.  (2018) (24)  Comparison of administration type of treatment | In vitro:  Tenocytes isolated from 8-week old Sprague Dawley(SD) Rats  In vivo:  28 male 8-week-old SD rats  Groups (n= 4 per group):  -Control (no treatment),  -Col (I) (collagenase treatment),  -Col (I) + PMSs,  -Col (I) + Cur (0.5 wt%)/PMSs,  -Col (I) + Cur (2 wt%)/PMSs,  -Col (I) + Cur (5 wt%)/PMSs, and  -Col (I) + Curcumin | In vitro  After 3 days  In vivo  After 7 weeks | In vitro:  Treated with lipopolysaccharides  In vivo:  Injection with collagenase type I 50 uL (50mg/ml) | In vitro:  Curcumin-loaded  porous PLGA (poly (D,L-lactide-co-glycolide)) microspheres (Cur/PMS) 10mg  In vivo:  Injection with:  -Cur (0.5 wt%)/PMSs, 1.03 mg  -Cur (2 wt%)/PMSs, 4.30 mg/rat  -Cur (5 wt%)/PMSs, 10.37 mg/rat  -Positive control, curcumin (35 mg/rat) | In vivo:  Cur/PMSs compared to other treatment and no treatment groups  -Better collagen fiber orientation and decrease the number of inflammatory cells dose-dependently in  Cur/PMS compared to no treatment  -Prevented collagen disruption  -Repaired collagen matrix organization in a dose-dependently manner  -Curcumin also had positive effects but Cur/PMS effects were better | In vitro:  Treatment compared to no treatment  -Decreased expression of MMP-3, MMP-13, COX-2, ADAMTS-5, IL-6, and TNF-a  -Cur (5 wt%)/PMS highest decrease in inflammatory markers  In vivo:  -Decreased expression of MMP-3, MMP-13, COX-2, ADAMTS-5, IL-6, and TNF-a  -Cur (5 wt%)/PMS highest decrease in inflammatory markers | In vivo:  -Cur(5 wt%)/PMS best tensile strength compared to other treatment and PMS groups |
| Chen et al.  (2014) (25)  Comparison of single treatment effect and combination of treatments | In vitro:  Tendon derived stem cells cultured  In vivo:  18 8-week-old Sprague Dawley rats  Groups (n=24 per group):  -PRP  -TDSC  -PRP with TDSC (PRTD)  -Tendinopathy control (Tend)  -Healthy control (Sham) | After 4 and 6 weeks | In vivo:  Injection with collagenase type I 250 UI | In vitro:  Treated with 10% PRP  In vivo:  Intratendinous injection with:  -Allogenic PRP 20uL  -Allogenic Tendon derived stem cells (TDSC) 20 uL  -Combination | In vivo  -Treatment with TDSC no effect on histopathological scores  PRP and PRTD compared to Tend  -Lower nuclear rounding scores  -Better fiber structure, arrangement, and inflammation scores  PRP compared to PRTD  -Slightly better scores regarding Fiber arrangement, structure, nuclear rounding, and inflammation | In vitro  -Increased tenocyte differentiation  -Better collagen type I density  -Involvement of FAK and ERK1/2 signaling  In vivo  PRTD compared to Tend and other treatment groups  -An increased expression of collagen type I, Scx, Tenascin C  -Decreased expression of Runx2, PPARy, and SOX9 | In vivo  PRTD compared to Tend  -Better maximum load at weeks 4 and 8  -Better stiffness at week 8  PRP compared to Tend  -Better maximum load at week 8  -Better stiffness at week 8  PRTD compared to PRP  -Better mean stiffness |

# Appendix F - In vitro studies

| Author & Year  Study design | In vitro model | Time of analysis | Method of inducting AT | Treatment | Histological outcome | Biochemical outcome, | Biomechanical outcome |
| --- | --- | --- | --- | --- | --- | --- | --- |
| Pedrozo Vieira et al.  (2018) (26)  In vitro study | Tenocytes isolated from Male Wistar rats  Groups:  -Control group  -TNF-a 24 h  -Glycine post 24 h: TNF-aþglycine 24 h;  -Glycine pre TNF-B administration  -TNF-a 48 h;  -Glycine post 48 h: TNF-aþglycine 48 h | After 4, 6, 24, and 48 h. | Administration of TNF-B 10 ng/mL | Administration of glycine 20mM | NA | Glycine treatment compared to TNF-B  -Decreased expression of collagen type I, MMP-2, and MMP-3 after 24 and 48h  -Decreased levels of TIMP-2  -An increased expression of TIMP-1 after 48 hr  -Less uronic acid content  Pre Glycine group compared to Normal  -Similar expression of collagen type I  -Lower MMP-2 levels  -Increased TIMP-2 levels  - MMP-9 was elevated in 48 h in comparison with 24 h | NA |
| Chen et al.  (2012) (27)  In vitro study | Tendon stem cells derived from 6-weeks old male Sprague Dawley rats (n=9)  Groups:  -PRP medium 2%  -PRP medium 10%  -Control medium | Up to 21 days | Cyclic 8%  stretching at 0.5 Hz was applied for 12 h | Medium supplemented  with 2% or 10% Platelet Rich Cloth Release | NR | 10% PRP compared to control  -Increased levels of collagen type I and III  -Decreased levels of adipocyte marker PPARy  -Decreased levels of chondrocyte marker SOX-9  -Decreased levels of RUNX2  -Decreased numbers of oil red O-positive TSC-derived adipocytes and Safranin O-positive TSC-derived osteocytes  -Increased levels of VEGF  -Increased TGN-B1 | NR |

References

1. Weiss E. Calcaneal spurs: examining etiology using prehistoric skeletal remains to understand present day heel pain. Foot (Edinb). 2012;22(3):125-9.

2. Li HY, Hua YH. Achilles Tendinopathy: Current Concepts about the Basic Science and Clinical Treatments. Biomed Res Int. 2016;2016:6492597.

3. Silbernagel KG, Hanlon S, Sprague A. Current Clinical Concepts: Conservative Management of Achilles Tendinopathy. J Athl Train. 2020;55(5):438-47.

4. van der Vlist AC, Winters M, Weir A, Ardern CL, Welton NJ, Caldwell DM, et al. Which treatment is most effective for patients with Achilles tendinopathy? A living systematic review with network meta-analysis of 29 randomised controlled trials. British Journal of Sports Medicine. 2021;55(5):249-56.

5. Tarantino D, Mottola R, Resta G, Gnasso R, Palermi S, Corrado B, et al. Achilles Tendinopathy Pathogenesis and Management: A Narrative Review. Int J Environ Res Public Health. 2023;20(17).

6. Maffulli N, Sharma P, Luscombe KL. Achilles tendinopathy: aetiology and management. J R Soc Med. 2004;97(10):472-6.

7. Knapik JJ, Pope R. Achilles Tendinopathy: Pathophysiology, Epidemiology, Diagnosis, Treatment, Prevention, and Screening. J Spec Oper Med. 2020;20(1):125-40.

8. Edgar N, Clifford C, O'Neill S, Pedret C, Kirwan P, Millar NL. Biopsychosocial approach to tendinopathy. BMJ Open Sport &amp; Exercise Medicine. 2022;8(3):e001326.

9. Millar NL, Murrell GAC, McInnes IB. Inflammatory mechanisms in tendinopathy – towards translation. Nature Reviews Rheumatology. 2017;13(2):110-22.

10. Lui PPY, Maffulli N, Rolf C, Smith RKW. What are the validated animal models for tendinopathy? Scandinavian Journal of Medicine & Science in Sports. 2011;21(1):3-17.

11. Diederich K, Schmitt K, Schwedhelm P, Bert B, Heinl C. A guide to open science practices for animal research. PLoS Biol. 2022;20(9):e3001810.

12. Mourad Ouzzani HH, Zbys Fedorowicz, and Ahmed Elmagarmid. Rayyan — a web and mobile app for systematic reviews Systematic Reviews. 2016; 5:210.

13. Hooijmans CR, Rovers MM, de Vries RBM, Leenaars M, Ritskes-Hoitinga M, Langendam MW. SYRCLE’s risk of bias tool for animal studies. BMC Medical Research Methodology. 2014;14(1):43.

14. McGuinness LA, Higgins JPT. Risk-of-bias VISualization (robvis): An R package and Shiny web app for visualizing risk-of-bias assessments. Research Synthesis Methods. 2020;n/a(n/a).

15. Lee JM, Hwang JW, Kim MJ, Jung SY, Kim KS, Ahn EH, et al. Mitochondrial Transplantation Modulates Inflammation and Apoptosis, Alleviating Tendinopathy Both In Vivo and In Vitro. Antioxidants (Basel). 2021;10(5).

16. Liu YC, Wang HL, Huang YZ, Weng YH, Chen RS, Tsai WC, et al. Alda-1, an activator of ALDH2, ameliorates Achilles tendinopathy in cellular and mouse models. Biochem Pharmacol. 2020;175:113919.

17. Ruan D, Fei Y, Qian S, Huang Z, Chen W, Tang C, et al. Early-Stage Primary Anti-inflammatory Therapy Enhances the Regenerative Efficacy of Platelet-Rich Plasma in a Rabbit Achilles Tendinopathy Model. Am J Sports Med. 2021:3635465211037354.

18. Al-Shudiefat AAS, Am Alzyoud J, Al Najjar SA, Talat S, Bustanji Y, Abu-Irmaileh B. The effects of some natural products compared to synthetic products on the metabolic activity, proliferation, viability, migration, and wound healing in sheep tenocytes. Saudi J Biol Sci. 2022;29(9):103391.

19. Choi HJ, Choi S, Kim JG, Song MH, Shim KS, Lim YM, et al. Enhanced tendon restoration effects of anti-inflammatory, lactoferrin-immobilized, heparin-polymeric nanoparticles in an Achilles tendinitis rat model. Carbohydr Polym. 2020;241:116284.

20. Choi S, Song MH, Shim KS, Kim HJ, Lim YM, Song HR, et al. Therapeutic Efficacy of Intratendinous Delivery of Dexamethasone Using Porous Microspheres for Amelioration of Inflammation and Tendon Degeneration on Achilles Tendinitis in Rats. Biomed Res Int. 2020;2020:5052028.

21. Wang Y, He G, Tang H, Shi Y, Zhu M, Kang X, et al. Aspirin promotes tenogenic differentiation of tendon stem cells and facilitates tendinopathy healing through regulating the GDF7/Smad1/5 signaling pathway. J Cell Physiol. 2020;235(5):4778-89.

22. Zhao G, Zhang J, Nie D, Zhou Y, Li F, Onishi K, et al. HMGB1 mediates the development of tendinopathy due to mechanical overloading. PLoS One. 2019;14(9):e0222369.

23. Jeong C, Kim SE, Shim KS, Kim HJ, Song MH, Park K, et al. Exploring the In Vivo Anti-Inflammatory Actions of Simvastatin-Loaded Porous Microspheres on Inflamed Tenocytes in a Collagenase-Induced Animal Model of Achilles Tendinitis. Int J Mol Sci. 2018;19(3).

24. Kim SE, Yun YP, Shim KS, Jeon DI, Park K, Kim HJ. In vitro and in vivo anti-inflammatory and tendon-healing effects in Achilles tendinopathy of long-term curcumin delivery using porous microspheres. JOURNAL OF INDUSTRIAL AND ENGINEERING CHEMISTRY. 2018;58:123-30.

25. Chen L, Liu JP, Tang KL, Wang Q, Wang GD, Cai XH, et al. Tendon derived stem cells promote platelet-rich plasma healing in collagenase-induced rat achilles tendinopathy. Cell Physiol Biochem. 2014;34(6):2153-68.

26. Vieira CP, Viola M, Carneiro GD, D'Angelo ML, Vicente CP, Passi A, et al. Glycine improves the remodeling process of tenocytes in vitro. Cell Biol Int. 2018;42(7):804-14.

27. Chen L, Dong SW, Tao X, Liu JP, Tang KL, Xu JZ. Autologous platelet-rich clot releasate stimulates proliferation and inhibits differentiation of adult rat tendon stem cells towards nontenocyte lineages. J Int Med Res. 2012;40(4):1399-409.

28. Zhang J, Li F, Nie D, Onishi K, Hogan MV, Wang JH. Effect of Metformin on Development of Tendinopathy Due to Mechanical Overloading in an Animal Model. Foot Ankle Int. 2020;41(12):1455-65.

29. Ng GY, Chung PY. Effects of a therapeutic laser and passive stretching program for treating tendon overuse. Photomed Laser Surg. 2012;30(3):155-9.

30. Imai K, Ikoma K, Chen Q, Zhao C, An KN, Gay RE. Biomechanical and histological effects of augmented soft tissue mobilization therapy on achilles tendinopathy in a rabbit model. J Manipulative Physiol Ther. 2015;38(2):112-8.

31. Çınar BM, Çirci E, Balçık C, Güven G, Akpınar S, Derincek A. The effects of extracorporeal shock waves on carrageenan-induced Achilles tendinitis in rats: a biomechanical and histological analysis. Acta Orthop Traumatol Turc. 2013;47(4):266-72.

32. Marcos RL, Leal Junior EC, Messias Fde M, de Carvalho MH, Pallotta RC, Frigo L, et al. Infrared (810 nm) low-level laser therapy in rat achilles tendinitis: a consistent alternative to drugs. Photochem Photobiol. 2011;87(6):1447-52.

33. Marsolais D, Côté CH, Frenette J. Nonsteroidal anti-inflammatory drug reduces neutrophil and macrophage accumulation but does not improve tendon regeneration. Lab Invest. 2003;83(7):991-9.

34. Godbout C, Ang O, Frenette J. Early voluntary exercise does not promote healing in a rat model of Achilles tendon injury. J Appl Physiol (1985). 2006;101(6):1720-6.

35. Tatari H, Koşay C, Baran O, Ozcan O, Ozer E, Ulukuş C. Effect of heparin on tendon degeneration: an experimental study on rats. Knee Surg Sports Traumatol Arthrosc. 2001;9(4):247-53.

36. Kim J, Seo BB, Hong KH, Kim SE, Kim YM, Song SC. Long-term anti-inflammatory effects of injectable celecoxib nanoparticle hydrogels for Achilles tendon regeneration. Acta Biomaterialia. 2022;144:183-94.

37. Gundogdu K, Tasci SY, Nalci KA, Gundogdu G, Kapakin KAT, Demirkaya AK, et al. Effects of Docosahexaenoic Acid (DHA, Omega 3) with Exercise on Experimental Achilles Tendinopathy in Rats. ACTA PHYSIOLOGICA. 2019;227:57-.

38. Ge Z, Li W, Zhao R, Xiong W, Wang D, Tang Y, et al. Programmable DNA Hydrogel Provides Suitable Microenvironment for Enhancing TSPCS Therapy in Healing of Tendinopathy. Small. 2023:e2207231.

39. Ma Y, Lin Z, Chen X, Zhao X, Sun Y, Wang J, et al. Human hair follicle-derived mesenchymal stem cells promote tendon repair in a rabbit Achilles tendinopathy model. Chin Med J (Engl). 2023.

40. Chen J, Yu Q, Wu B, Lin Z, Pavlos NJ, Xu J, et al. Autologous tenocyte therapy for experimental Achilles tendinopathy in a rabbit model. Tissue Eng Part A. 2011;17(15):2037-48.

41. Jiang H, Lin X, Liang W, Li Y, Yu X. Friedelin Alleviates the Pathogenesis of Collagenase-Induced Tendinopathy in Mice by Promoting the Selective Autophagic Degradation of p65. Nutrients. 2022;14(8).

42. Vieira CP, Guerra Fda R, de Oliveira LP, Almeida MS, Marcondes MC, Pimentell ER. Green tea and glycine aid in the recovery of tendinitis of the Achilles tendon of rats. Connect Tissue Res. 2015;56(1):50-8.

43. Lee TH, Kim SE, Lee JY, Kim JG, Park K, Kim HJ. Wrapping of tendon tissues with diclofenac-immobilized polycaprolactone fibrous sheet improves tendon healing in a rabbit model of collagenase-induced Achilles tendinitis. JOURNAL OF INDUSTRIAL AND ENGINEERING CHEMISTRY. 2019;73:152-61.

44. Ko KR, Han SH, Choi S, An HJ, Kwak EB, Jeong Y, et al. Substance P Inhibitor Promotes Tendon Healing in a Collagenase-Induced Rat Model of Tendinopathy. Am J Sports Med. 2022;50(13):3681-9.

45. Gundogdu G, Tasci SY, Gundogdu K, Kapakin KAT, Demirkaya AK, Nalci KA, et al. A combination of omega-3 and exercise reduces experimental Achilles tendinopathy induced with a type-1 collagenase in rats. Appl Physiol Nutr Metab. 2023;48(1):62-73.

46. Wu Y, Qian J, Li K, Li W, Yin W, Jiang H. Farrerol alleviates collagenase-induced tendinopathy by inhibiting ferroptosis in rats. J Cell Mol Med. 2022;26(12):3483-94.

47. Oshita T, Tobita M, Tajima S, Mizuno H. Adipose-Derived Stem Cells Improve Collagenase-Induced Tendinopathy in a Rat Model. Am J Sports Med. 2016;44(8):1983-9.

48. Lacitignola L, Staffieri F, Rossi G, Francioso E, Crovace A. Survival of bone marrow mesenchymal stem cells labelled with red fluorescent protein in an ovine model of collagenase-induced tendinitis. Vet Comp Orthop Traumatol. 2014;27(3):204-9.

49. Jiang G, Wu Y, Meng J, Wu F, Li S, Lin M, et al. Comparison of Leukocyte-Rich Platelet-Rich Plasma and Leukocyte-Poor Platelet-Rich Plasma on Achilles Tendinopathy at an Early Stage in a Rabbit Model. Am J Sports Med. 2020;48(5):1189-99.

50. Marcos RL, Leal-Junior EC, Arnold G, Magnenet V, Rahouadj R, Wang X, et al. Low-level laser therapy in collagenase-induced Achilles tendinitis in rats: analyses of biochemical and biomechanical aspects. J Orthop Res. 2012;30(12):1945-51.

51. Vieira CP, De Oliveira LP, Da Ré Guerra F, Dos Santos De Almeida M, Marcondes MC, Pimentel ER. Glycine improves biochemical and biomechanical properties following inflammation of the achilles tendon. Anat Rec (Hoboken). 2015;298(3):538-45.

52. Solchaga LA, Bendele A, Shah V, Snel LB, Kestler HK, Dines JS, et al. Comparison of the effect of intra-tendon applications of recombinant human platelet-derived growth factor-BB, platelet-rich plasma, steroids in a rat achilles tendon collagenase model. J Orthop Res. 2014;32(1):145-50.

53. Bell R, Li J, Gorski DJ, Bartels AK, Shewman EF, Wysocki RW, et al. Controlled treadmill exercise eliminates chondroid deposits and restores tensile properties in a new murine tendinopathy model. J Biomech. 2013;46(3):498-505.

54. Emrani H, Davies JE. Umbilical cord perivascular cells: A mesenchymal cell source for treatment of tendon injuries. Open Tissue Engineering and Regenerative Medicine Journal. 2011;4:112-9.

55. Dallaudière B, Lempicki M, Pesquer L, Louedec L, Preux PM, Meyer P, et al. Efficacy of intra-tendinous injection of platelet-rich plasma in treating tendinosis: comprehensive assessment of a rat model. Eur Radiol. 2013;23(10):2830-7.

56. Li S, Wu Y, Jiang G, Tian X, Hong J, Chen S, et al. Intratendon delivery of leukocyte-rich platelet-rich plasma at early stage promotes tendon repair in a rabbit Achilles tendinopathy model. J Tissue Eng Regen Med. 2020;14(3):452-63.

57. Sánchez‐sánchez JL, Calderón‐díez L, Herrero‐turrión J, Méndez‐sánchez R, Arias‐buría JL, Fernández‐de‐las‐peñas C. Changes in gene expression associated with collagen regeneration and remodeling of extracellular matrix after percutaneous electrolysis on collagenase‐induced achilles tendinopathy in an experimental animal model: A pilot study. Journal of Clinical Medicine. 2020;9(10):1-17.

58. Xavier M, David DR, de Souza RA, Arrieiro AN, Miranda H, Santana ET, et al. Anti-inflammatory effects of low-level light emitting diode therapy on Achilles tendinitis in rats. Lasers Surg Med. 2010;42(6):553-8.

59. Pires D, Xavier M, Araujo T, Silva JA, Aimbire F, Albertini R. Low-level laser therapy (LLLT; 780 nm) acts differently on mRNA expression of anti- and pro-inflammatory mediators in an experimental model of collagenase-induced tendinitis in rat. LASERS IN MEDICAL SCIENCE. 2011;26(1):85-94.

60. Fedato RA, Francisco JC, Sliva G, de Noronha L, Olandoski M, Faria Neto JR, et al. Stem Cells and Platelet-Rich Plasma Enhance the Healing Process of Tendinitis in Mice. Stem Cells Int. 2019;2019:1497898.

61. Johnson SA, Sikes KJ, Thampi P, McConnell A, Coghlan R, Johnstone B, et al. Fast, non-eccentrically loaded exercise worsens tendinopathic healing responses in a murine model. Am J Vet Res. 2023:1-9.

62. Evangelista AN, Dos Santos FF, de Oliveira Martins LP, Gaiad TP, Machado ASD, Rocha-Vieira E, et al. Photobiomodulation therapy on expression of HSP70 protein and tissue repair in experimental acute Achilles tendinitis. Lasers Med Sci. 2021;36(6):1201-8.

63. Xavier M, de Souza RA, Pires VA, Santos AP, Aimbire F, Silva JA, Jr., et al. Low-level light-emitting diode therapy increases mRNA expressions of IL-10 and type I and III collagens on Achilles tendinitis in rats. Lasers Med Sci. 2014;29(1):85-90.

64. Naterstad IF, Rossi RP, Marcos RL, Parizzoto NA, Frigo L, Joensen J, et al. Comparison of Photobiomodulation and Anti-Inflammatory Drugs on Tissue Repair on Collagenase-Induced Achilles Tendon Inflammation in Rats. Photomed Laser Surg. 2018;36(3):137-45.

65. Guerra FD, Vieira CP, Marques PP, Oliveira LP, Pimentel ER. Low level laser therapy accelerates the extracellular matrix reorganization of inflamed tendon. TISSUE & CELL. 2017;49(4):483-8.

66. Marques AC, Albertini R, Serra AJ, da Silva EA, de Oliveira VL, Silva LM, et al. Photobiomodulation therapy on collagen type I and III, vascular endothelial growth factor, and metalloproteinase in experimentally induced tendinopathy in aged rats. Lasers Med Sci. 2016;31(9):1915-23.

67. Torres-Silva R, Lopes-Martins RA, Bjordal JM, Frigo L, Rahouadj R, Arnold G, et al. The low level laser therapy (LLLT) operating in 660 nm reduce gene expression of inflammatory mediators in the experimental model of collagenase-induced rat tendinitis. Lasers Med Sci. 2015;30(7):1985-90.

68. Marcos RL, Arnold G, Magnenet V, Rahouadj R, Magdalou J, Lopes-Martins R. Biomechanical and biochemical protective effect of low-level laser therapy for Achilles tendinitis. J Mech Behav Biomed Mater. 2014;29:272-85.

69. Casalechi HL, Leal ECP, Xavier M, Silva JA, de Tarso P, de Carvalho C, et al. Low-level laser therapy in experimental model of collagenase-induced tendinitis in rats: effects in acute and chronic inflammatory phases. LASERS IN MEDICAL SCIENCE. 2013;28(3):989-95.

70. Chen YJ, Wang CJ, Yang KD, Kuo YR, Huang HC, Huang YT, et al. Extracorporeal shock waves promote healing of collagenase-induced Achilles tendinitis and increase TGF-beta1 and IGF-I expression. J Orthop Res. 2004;22(4):854-61.

71. Tsai YP, Chang CW, Lee JS, Liang JI, Hsieh TH, Yeh ML, et al. Direct radiofrequency application improves pain and gait in collagenase-induced acute achilles tendon injury. Evid Based Complement Alternat Med. 2013;2013:402692.

72. Naseri F, Shahri NM, Sardari K, Moghimi A, Bahrami AR, Kheirabadi M, et al. Experimental study of the tendon healing and remodeling after local injection of bone marrow myeloid tissue in rabbit. Journal of Biological Sciences. 2008;8(3):591-7.

73. Martins M, Maia Filho AL, Costa CL, Coelho NP, Costa MS, Carvalho RA. Anti-inflammatory action of the Ovis aries lipidic fraction associated to therapeutic ultrasound in an experimental model of tendinitis in rats (Rattus norvegicus). Rev Bras Fisioter. 2011;15(4):297-302.

74. Perucca Orfei C, Lovati AB, Lugano G, Viganò M, Bottagisio M, D'Arrigo D, et al. Pulsed electromagnetic fields improve the healing process of Achilles tendinopathy: a pilot study in a rat model. Bone Joint Res. 2020;9(9):613-22.

75. Facon-Poroszewska M, Kiełbowicz Z, Prządka P. Influence of Radial Pressure Wave Therapy (RPWT) on collagenase-induced Achilles tendinopathy treated with Platelet Rich Plasma and Autologous Adipose Derived Stem Cells. Pol J Vet Sci. 2019;22(4):743-51.

76. Kamineni S, Butterfield T, Sinai A. Percutaneous ultrasonic debridement of tendinopathy-a pilot Achilles rabbit model. J Orthop Surg Res. 2015;10:70.

77. Gehlsen GM, Ganion LR, Helfst R. Fibroblast responses to variation in soft tissue mobilization pressure. Med Sci Sports Exerc. 1999;31(4):531-5.

78. Davidson CJ, Ganion LR, Gehlsen GM, Verhoestra B, Roepke JE, Sevier TL. Rat tendon morphologic and functional changes resulting from soft tissue mobilization. Med Sci Sports Exerc. 1997;29(3):313-9.

79. Zhang J, Pan T, Wang JH. Cryotherapy suppresses tendon inflammation in an animal model. J Orthop Translat. 2014;2(2):75-81.

80. Yan R, Gu Y, Ran J, Hu Y, Zheng Z, Zeng M, et al. Intratendon Delivery of Leukocyte-Poor Platelet-Rich Plasma Improves Healing Compared With Leukocyte-Rich Platelet-Rich Plasma in a Rabbit Achilles Tendinopathy Model. Am J Sports Med. 2017;45(8):1909-20.

81. Dallaudiere B, Louedec L, Lenet MP, Pesquer L, Blaise E, Perozziello A, et al. The molecular systemic and local effects of intra-tendinous injection of Platelet Rich Plasma in tendinosis: preliminary results on a rat model with ELISA method. Muscles Ligaments Tendons J. 2015;5(2):99-105.

82. González JC, López C, Álvarez ME, Pérez JE, Carmona JU. Autologous leukocyte-reduced platelet-rich plasma therapy for Achilles tendinopathy induced by collagenase in a rabbit model. Sci Rep. 2016;6:19623.

83. Calandruccio JH, Cannon TA, Wodowski AJ, Stephens BF, Smith RA. A mechanical and histologic comparative study of the effect of saline, steroid, autologous blood, and platelet-rich plasma on collagenase-induced Achilles tendinopathy in a rat model. Current Orthopaedic Practice. 2015;26(6):E7-E12.

84. Dallaudiere B, Zurlinden O, Perozziello A, Deschamps L, Larbi A, Louedec L, et al. Combined intra-tendinous injection of Platelet Rich Plasma and bevacizumab accelerates and improves healing compared to Platelet Rich Plasma in tendinosis: comprehensive assessment on a rat model. Muscles Ligaments Tendons J. 2014;4(3):351-6.

85. Palumbo Piccionello A, Riccio V, Senesi L, Volta A, Pennasilico L, Botto R, et al. Adipose micro-grafts enhance tendinopathy healing in ovine model: An in vivo experimental perspective study. Stem Cells Transl Med. 2021.

86. Kokubu S, Inaki R, Hoshi K, Hikita A. Adipose-derived stem cells improve tendon repair and prevent ectopic ossification in tendinopathy by inhibiting inflammation and inducing neovascularization in the early stage of tendon healing. Regen Ther. 2020;14:103-10.

87. Chen QJ, Chen L, Wu SK, Wu YJ, Pang QJ. rhPDGF-BB combined with ADSCs in the treatment of Achilles tendinitis via miR-363/PI3 K/Akt pathway. Mol Cell Biochem. 2018;438(1):175-82.

88. Rezvani SN, Chen J, Li J, Midura R, Cali V, Sandy JD, et al. In-Vivo Efficacy of Recombinant Human Hyaluronidase (rHuPH20) Injection for Accelerated Healing of Murine Retrocalcaneal Bursitis and Tendinopathy. J Orthop Res. 2020;38(1):59-69.

89. de Girolamo L, Morlin Ambra LF, Perucca Orfei C, McQuilling JP, Kimmerling KA, Mowry KC, et al. Treatment with Human Amniotic Suspension Allograft Improves Tendon Healing in a Rat Model of Collagenase-Induced Tendinopathy. Cells. 2019;8(11).

90. Ahrberg AB, Horstmeier C, Berner D, Brehm W, Gittel C, Hillmann A, et al. Effects of mesenchymal stromal cells versus serum on tendon healing in a controlled experimental trial in an equine model. BMC MUSCULOSKELETAL DISORDERS. 2018;19.

91. Machova Urdzikova L, Sedlacek R, Suchy T, Amemori T, Ruzicka J, Lesny P, et al. Human multipotent mesenchymal stem cells improve healing after collagenase tendon injury in the rat. Biomed Eng Online. 2014;13:42.

92. Crovace A, Lacitignola L, Francioso E, Rossi G. Histology and immunohistochemistry study of ovine tendon grafted with cBMSCs and BMMNCs after collagenase-induced tendinitis. Vet Comp Orthop Traumatol. 2008;21(4):329-36.

93. Shah V, Bendele A, Dines JS, Kestler HK, Hollinger JO, Chahine NO, et al. Dose-response effect of an intra-tendon application of recombinant human platelet-derived growth factor-BB (rhPDGF-BB) in a rat Achilles tendinopathy model. J Orthop Res. 2013;31(3):413-20.

94. Watts AE, Millar NL, Platt J, Kitson SM, Akbar M, Rech R, et al. MicroRNA29a Treatment Improves Early Tendon Injury. MOLECULAR THERAPY. 2017;25(10):2415-26.

95. Xu XQ, Wang RL, Li YX, Wu R, Yan WJ, Zhao S, et al. Cerium oxide nanozymes alleviate oxidative stress in tenocytes for Achilles tendinopathy healing. NANO RESEARCH. 2023.

96. Micheli L, Parisio C, Lucarini E, Carrino D, Ciampi C, Toti A, et al. Restorative and pain-relieving effects of fibroin in preclinical models of tendinopathy. BIOMEDICINE & PHARMACOTHERAPY. 2022;148.

97. Yamaura K, Mifune Y, Inui A, Nishimoto H, Kurosawa T, Mukohara S, et al. Antioxidant effect of nicotinamide mononucleotide in tendinopathy. BMC Musculoskelet Disord. 2022;23(1):249.

98. Dallaudière B, Lempicki M, Pesquer L, Louedec L, Preux PM, Meyer P, et al. Acceleration of tendon healing using US guided intratendinous injection of bevacizumab: first pre-clinical study on a murine model. Eur J Radiol. 2013;82(12):e823-8.

99. Ueda H, Meguri N, Minaguchi J, Watanabe T, Nagayasu A, Hosaka Y, et al. Effect of collagen oligopeptide injection on rabbit tenositis. J Vet Med Sci. 2008;70(12):1295-300.

100. Williams IF, Nicholls JS, Goodship AE, Silver IA. Experimental treatment of tendon injury with heparin. Br J Plast Surg. 1986;39(3):367-72.

101. Yamamoto E, Hata D, Kobayashi A, Ueda H, Tangkawattana P, Oikawa M, et al. Effect of beta-aminopropionitrile and hyaluronic acid on repair of collagenase-induced injury of the rabbit Achilles tendon. J Comp Pathol. 2002;126(2):161-70.

102. Jiao X, Wang Z, Li Y, Wang T, Xu C, Zhou X, et al. Fullerenol inhibits tendinopathy by alleviating inflammation. Front Bioeng Biotechnol. 2023;11:1171360.

103. Tatari H, Skiak E, Destan H, Ulukuş C, Ozer E, Satoğlu S. Effect of hylan G-F 20 in Achilles' tendonitis: an experimental study in rats. Arch Phys Med Rehabil. 2004;85(9):1470-4.

104. Wang K, Cheng L, He B. Therapeutic effects of asperosaponin VI in rabbit tendon disease. Regen Ther. 2022;20:1-8.

105. Hsieh YL, Lin MT, Hong CZ, Chen HS. Percutaneous soft tissue release performed using a blunt cannula in rabbits with chronic collagenase-induced Achilles tendinopathy. Foot Ankle Surg. 2019;25(2):186-92.

106. Wang X, Xu K, Zhang E, Bai Q, Ma B, Zhao C, et al. Irreversible Electroporation Improves Tendon Healing in a Rat Model of Collagenase-Induced Achilles Tendinopathy. Am J Sports Med. 2023:3635465231167860.

107. Gunes T, Bilgic E, Erdem M, Bostan B, Koseoglu RD, Sahin SA, et al. Effect of radiofrequency microtenotomy on degeneration of tendons: an experimental study on rabbits. Foot Ankle Surg. 2014;20(1):61-6.

108. Vieira CP, De Oliveira LP, Da Ré Guerra F, Marcondes MC, Pimentel ER. Green Tea and Glycine Modulate the Activity of Metalloproteinases and Collagen in the Tendinitis of the Myotendinous Junction of the Achilles Tendon. Anat Rec (Hoboken). 2016;299(7):918-28.

109. Bittermann A, Gao S, Rezvani S, Li J, Sikes KJ, Sandy J, et al. Oral Ibuprofen Interferes with Cellular Healing Responses in a Murine Model of Achilles Tendinopathy. J Musculoskelet Disord Treat. 2018;4(2).

110. Gong F, Cui L, Zhang X, Zhan X, Gong X, Wen Y. Piperine ameliorates collagenase-induced Achilles tendon injury in the rat. Connect Tissue Res. 2018;59(1):21-9.

111. Gundogdu K, Yilmaz Tasci S, Gundogdu G, Terim Kapakin KA, Totik Y, Demirkaya Miloglu F. Evaluation of cytokines in protective effect of docosahexaenoic acid in experimental achilles tendinopathy rat model induced with type-1 collagenase. Connective Tissue Research. 2021:1-13.

112. Semis HS, Gur C, Ileriturk M, Kandemir FM, Kaynar O. Evaluation of Therapeutic Effects of Quercetin Against Achilles Tendinopathy in Rats via Oxidative Stress, Inflammation, Apoptosis, Autophagy, and Metalloproteinases. Am J Sports Med. 2022;50(2):486-98.

113. Xu T, Lin Y, Yu X, Jiang G, Wang J, Xu K, et al. Comparative Effects of Exosomes and Ectosomes Isolated From Adipose-Derived Mesenchymal Stem Cells on Achilles Tendinopathy in a Rat Model. Am J Sports Med. 2022;50(10):2740-52.

114. Yoo SD, Choi S, Lee GJ, Chon J, Jeong YS, Park HK, et al. Effects of extracorporeal shockwave therapy on nanostructural and biomechanical responses in the collagenase-induced Achilles tendinitis animal model. Lasers Med Sci. 2012;27(6):1195-204.

115. Leal SS, Uchoa VT, Figueredo-Silva J, Soares RB, Mota DM, de Alencar RC, et al. PHONOPHORESIS EFFECTIVENESS WITH XIMENIA AMERICANA L. IN RATS TENDON INFLAMMATION. REVISTA BRASILEIRA DE MEDICINA DO ESPORTE. 2016;22(5):355-60.

116. Wu PT, Jou IM, Kuo LC, Su FC. Intratendinous Injection of Hyaluronate Induces Acute Inflammation: A Possible Detrimental Effect. PLoS One. 2016;11(5):e0155424.

117. Perucca Orfei C, Lovati AB, Viganò M, Stanco D, Bottagisio M, Di Giancamillo A, et al. Dose-Related and Time-Dependent Development of Collagenase-Induced Tendinopathy in Rats. PLoS One. 2016;11(8):e0161590.

118. Warden SJ. Animal models for the study of tendinopathy. Br J Sports Med. 2007;41(4):232-40.

119. Zhang G, Zhou X, Hu S, Jin Y, Qiu Z. Large animal models for the study of tendinopathy. Frontiers in Cell and Developmental Biology. 2022;10.

120. Birch HL. Tendon matrix composition and turnover in relation to functional requirements. Int J Exp Pathol. 2007;88(4):241-8.

121. Tondelli T, Gotschi T, Camenzind RS, Snedeker JG. Assessing the effects of intratendinous genipin injections: Mechanical augmentation and spatial distribution in an ex vivo degenerative tendon model. PLOS ONE. 2020;15(4).

122. Martin RL, Chimenti R, Cuddeford T, Houck J, Matheson JW, McDonough CM, et al. Achilles Pain, Stiffness, and Muscle Power Deficits: Midportion Achilles Tendinopathy Revision 2018. Journal of Orthopaedic & Sports Physical Therapy. 2018;48(5):A1-A38.

123. de Vos R-J, van der Vlist AC, Zwerver J, Meuffels DE, Smithuis F, van Ingen R, et al. Dutch multidisciplinary guideline on Achilles tendinopathy. British Journal of Sports Medicine. 2021:bjsports-2020-103867.

124. Sterne JAC, Savović J, Page MJ, Elbers RG, Blencowe NS, Boutron I, et al. RoB 2: a revised tool for assessing risk of bias in randomised trials. BMJ. 2019;366:l4898.

125. Waffenschmidt S, Knelangen M, Sieben W, Bühn S, Pieper D. Single screening versus conventional double screening for study selection in systematic reviews: a methodological systematic review. BMC Medical Research Methodology. 2019;19(1):132.
